# Past, present, and future of global research on artificial intelligence applications in dermatology: A bibliometric analysis

**DOI:** 10.1097/MD.0000000000035993

**Published:** 2023-11-10

**Authors:** Guangxin Wang, Xianguang Meng, Fan Zhang

**Affiliations:** a Shandong Innovation Center of Intelligent Diagnosis, Jinan Central Hospital, Shandong University, Jinan, Shandong, China; b Department of Dermatology, Jinan Central Hospital, Shandong University, Jinan, Shandong, China.

**Keywords:** artificial intelligence, bibliometric analysis, deep learning, dermatology, machine learning, melanoma

## Abstract

In recent decades, artificial intelligence (AI) has played an increasingly important role in medicine, including dermatology. Worldwide, numerous studies have reported on AI applications in dermatology, rapidly increasing interest in this field. However, no bibliometric studies have been conducted to evaluate the past, present, or future of this topic. This study aimed to illustrate past and present research and outline future directions for global research on AI applications in dermatology using bibliometric analysis. We conducted an online search of the Web of Science Core Collection database to identify scientific papers on AI applications in dermatology. The bibliometric metadata of each selected paper were extracted, analyzed, and visualized using VOS viewer and Cite Space. A total of 406 papers, comprising 8 randomized controlled trials and 20 prospective studies, were deemed eligible for inclusion. The United States had the highest number of papers (n = 166). The University of California System (n = 24) and Allan C. Halpern (n = 11) were the institution and author with the highest number of papers, respectively. Based on keyword co-occurrence analysis, the studies were categorized into 9 distinct clusters, with clusters 2, 3, and 7 containing keywords with the latest average publication year. Wound progression prediction using machine learning, the integration of AI into teledermatology, and applications of the algorithms in skin diseases, are the current research priorities and will remain future research aims in this field.

## 1. Introduction

Dermatological diseases are currently the 4th most prevalent nonfatal diseases worldwide.^[1]^ The approach to the diagnosis and management of dermatological conditions has changed considerably with the advent of advanced technologies and inventions. Dermoscopy has improved the sensitivity and specificity of skin disease diagnoses; however, its diagnostic accuracy remains low. Moreover, 3 major challenges require resolution. First, the large amount of patient data, including clinical images, patient histories, and even genetic information, poses a distinctive challenge to dermatologists.^[2]^ Manual analysis of these data requires trained professionals to complete them, which is time-consuming, labor-intensive, and error-prone. Secondly, there is a serious shortage and uneven distribution of dermatologists, especially in developing countries and remote areas that urgently require additional medical facilities, professional consultations, and clinical assistance.^[3]^ Third, only 25% of melanomas were diagnosed by a healthcare provider.^[4]^ Therefore, the integration of artificial intelligence (AI) has immense potential for facilitating early evaluation, enhancing diagnostic precision, and optimizing treatment strategies, among other benefits.

AI is a computer science discipline that aims to simulate human cognitive functions such as learning, reasoning, communication, and decision-making.^[5,6]^ With the advancement of AI technologies such as deep learning and computer vision, AI has been widely used in various biomedical domains, including surgery, pediatrics, and dermatology.^[7–11]^ Many scholarly articles have been published on AI applications in dermatology, with increased interest in this field. Researchers began attempting to use expert system technologies to diagnose skin diseases in the 1980s and eventually developed a new system to promote the differential diagnosis of skin diseases.^[12]^ Nasr-Esfahani et al^[13]^ initially trained a neural network to identify melanomas, and their proposed method exhibited remarkable sensitivity and specificity. Recent studies have demonstrated that AI algorithms can diagnose skin diseases, especially skin cancer, with diagnostic accuracy comparable to that of dermatologists.^[14,15]^

The application of AI in dermatology involves the treatment of dermatological conditions. Robotic technology is currently being applied in skin surgery. According to a previous study, a robot-assisted automatic laser hair removal system outperformed physician-directed laser hair removal in terms of efficacy and safety.^[16]^ An automated robotic hair restoration device was introduced for clinical application in 2011, and its use has gained momentum worldwide as an alternative to manual follicular unit extraction.^[17–19]^ Additionally, AI systems can provide outcome predictions and prognostic assessments for dermatological diseases.^[20,21]^ Yeong et al^[22]^ developed an artificial neural network-based algorithm to predict when a burn would heal, with an overall prediction accuracy of 86%. Pivneva et al^[23]^ recently developed a prediction model to estimate the clinical remission time of patients with chronic urticaria using machine learning methods.

Many papers have been published on the applications of AI in dermatology. However, a global evaluation of knowledge frameworks, development trends, research hotspots, and future research directions is lacking. Bibliometric analysis is a suitable approach to study the global research landscape of AI applications in dermatology. This approach differs from traditional literature retrieval and review; it serves as an information visualization tool, facilitating the comprehension of research hotspots and their evolution, and predicting future research directions within the field.^[24,25]^ Using this method, we can identify the most active researchers, institutions, journals, influential papers, and international collaborations.

Several bibliometric studies have investigated AI applications in medical fields, such as cancer, diabetic retinopathy, critical care medicine, and cardiovascular diseases.^[26–31]^ However, no bibliometric analyses have been performed on AI applications in dermatology. Accordingly, this study aimed to illustrate past and present research and outline future directions for global research on AI applications in dermatology using bibliometric analysis.

## 2. Materials and methods

### 2.1. Data source and search strategy

**Figure 1.**
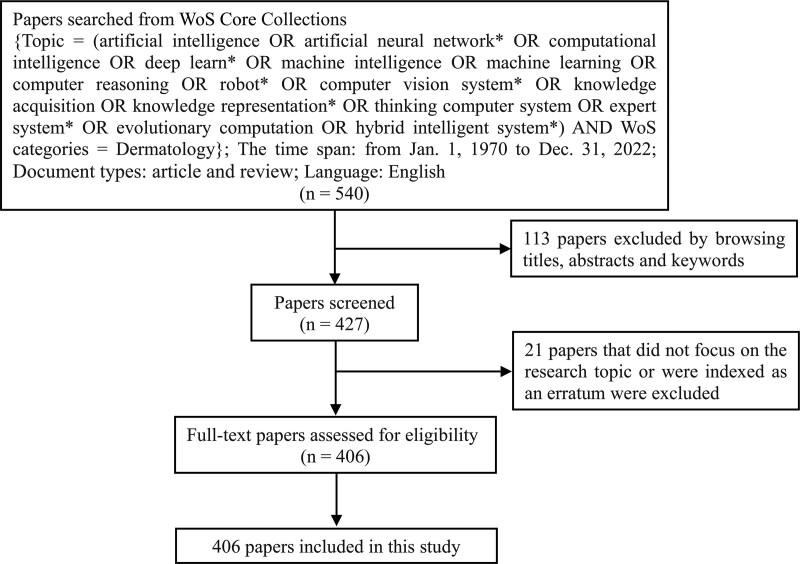
Flowchart depicting the paper selection process. The flowchart shows the search approach and criteria employed to identify papers on the applications of AI in dermatology from Web of Science core collection. AI = artificial intelligence.

On March 8, 2023, the Web of Science (WoS) Core Collection database provided by Thomson Reuters (Philadelphia, PA; https://www.webofscience.com/wos/alldb/basic-search) was searched for papers on AI applications in dermatology. The search strategy was as follows: (topic = [artificial intelligence OR artificial neural network* OR computational intelligence OR deep learning* OR machine intelligence OR machine learning OR computer reasoning OR robot* OR computer vision system* OR knowledge acquisition OR knowledge representation* OR thinking computer system OR expert system* OR evolutionary computation OR hybrid intelligent system*] AND WoS category = dermatology). The time interval of this study was 53 years, between January 1, 1970, and December 31, 2022, when medical researchers first discovered the applicability of AI in life sciences in the early 1970s.^[32]^ The document types were restricted to “article” and “review,” and the publications language was limited to English. Unrelated papers were excluded after carefully reviewing the titles, abstracts, and keywords. A total of 406 papers were included in this study. The authors names, affiliated institutions and countries, and any associated keywords in the 406 papers were subjected to unification and standardization. A flowchart depicting the paper selection process is shown in Figure 1. The full records of 406 papers were exported and saved in Microsoft Excel 2019 and End Note Desktop.

### 2.2. Ethical considerations

The Ethics Committee did not need to approve this study because it was a retrospective bibliometric analysis of previously published papers.

### 2.3. Data analysis and visualization

General information from the included papers was analyzed, and histograms and tables depicting the top 10 authors, institutions, countries, and journals, as well as the top 10 most-cited papers, were generated using Microsoft Excel 2019.

Visual analysis, including co-authorship and keyword co-occurrence analyses, was conducted using VOS viewer (version 1.6.19).

A keyword emergence analysis to detect burstness keywords was performed using the burstness function of Cite Space (version 6.2. R4).

## 3. Results

### 3.1. Annual published paper outputs and citations

**Figure 2.**
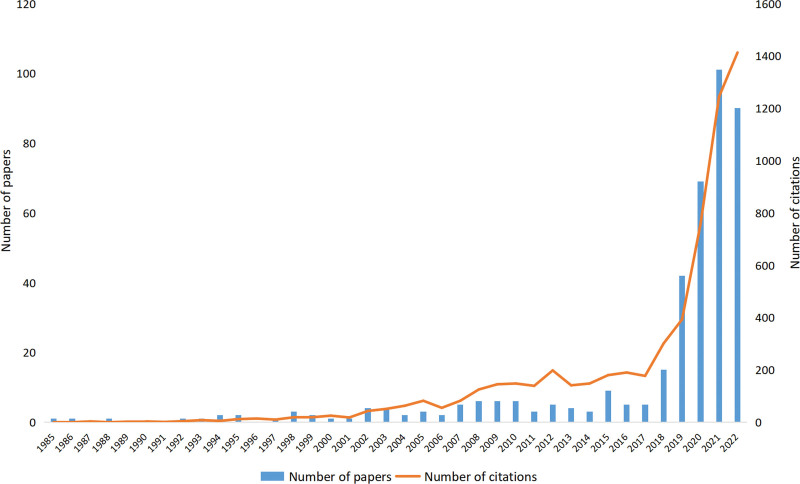
Trends in the annual number of published papers and citations.

We identified 406 papers on the topic of AI research in dermatology by searching WoS Core Collection (Supplemental Digital Content S1, http://links.lww.com/MD/K670); 348 (85.7%) were indexed as “articles” and 58 (14.3%) as “reviews.” The articles included only 8 randomized controlled trials and 20 prospective studies. The oldest study of this topic was published in 1985. Figure 2 shows the annual number of published papers and the citation trends. Before 2017, the number of annual papers in this field had grown slowly, with only 9 published annually at most, suggesting that research in this field remained in its infancy. However, after 2018, the number of papers increased rapidly, reaching 101 by 2021, which was more than 20 times the volume published in 2017. The cumulative number of papers published over the previous 5 years accounted for 78.1% of all papers. The number of annual citations increased steadily, although some fluctuations remained (Fig. 2).

### 3.2. Contributions of journals

Seventy-two journals had published 406 papers on AI applications in dermatology. Among these journals, 19 (26.4%) published only 1 paper. Table 1 presents the top 10 dermatology journals based on the number of published papers related to AI. When 2 journals had the same number of papers, the journal with the highest number of citations was ranked higher. Skin Research and Technology was the most productive journal, with 38 papers (9.4% of the total), followed by *The Journal of the European Academy of Dermatology and Venereology* (28, 6.9%). *The Journal of the American Academy of Dermatology* had the highest number of citations (n = 885). The impact factors of these journals in 2022 range from 2.189 to 15.487. According to the Cite Score 2021 report, 7 of the top 10 journals had Cite Scores > 5.

**Table 1:** The top 10 journals with the highest production in publishing papers concerning AI applications in dermatology.

Rank	Journals	Number of papers	Citations	2022 IF*	2021 Cite Score
1	*Skin Research and Technology*	38	452	2.2	3.5
2	*Journal of the European Academy of Dermatology and Venereology*	28	270	9.2	9.1
3	*Journal of the American Academy of Dermatology*	21	885	13.8	9.6
4	*Journal of Investigative Dermatology*	20	672	6.5	8.1
5	*Lasers in Surgery and Medicine*	17	234	2.4	5.2
6	*International Wound Journal*	17	78	3.1	5.1
7	*Burns*	15	248	2.7	3.9
8	*British Journal of Dermatology*	14	790	10.3	13.6
9	*Melanoma Research*	14	412	2.2	6.7
10	*Journal of Cosmetic Dermatology*	11	19	2.3	3.1

AI = artificial intelligence.

* Impact factor (IF) is derived from the journal citation report (2022).

### 3.3. Top 10 productive authors, institutions, and countries/regions

Allan C. Halpern had the highest number of papers published (n = 11) in this field, followed by Frederic Flament (n = 10) and Philipp Tschandl (n = 9) (Table 2). The University of California System emerged as the leading institution, with the highest number of papers (n = 24), followed by the Memorial Sloan Kettering Cancer Center (n = 23) (Table 3). Regarding countries/regions (Table 4), the United States ranked first (n = 166), followed by the United Kingdom (n = 33) and Germany (n = 31).

**Table 2:** The top 10 most productive authors ranked by the number of their published papers concerning AI in dermatology.

Rank	Author	Number of papers	Citations
1	Halpern AC	11	398
2	Flament F	10	50
3	Tschandl P	9	178
4	Gefen, A	8	20
5	Hofmann-Wellenhof R	7	276
6	Jiang RW	7	26
7	Soyer, HP	7	24
8	Rotemberg V	6	39
9	Goldust M	6	36
10	Delaunay, C	6	17

AI = artificial intelligence.

**Table 3:** The top 10 most productive institutions ranked by the number of their published papers concerning AI in dermatology.

Rank	Institution	Number of papers	Citations
1	The University of California System	24	339
2	Memorial Sloan Kettering Cancer Center	23	670
3	Harvard University	14	619
4	Tel Aviv University	14	208
5	University of Queensland	13	134
6	Icahn School of Medicine at Mount Sinai	13	92
7	Medical University of Vienna	12	259
8	New York University	11	549
9	Harvard Medical School	11	535
10	Stanford University	11	191

AI = artificial intelligence.

**Table 4:** The top 10 most productive countries/regions ranked by the number of their published papers concerning AI in dermatology.

Rank	Country/region	Number of papers	Citations
1	USA	166	2702
2	United Kingdom	33	394
3	Germany	31	759
4	China	31	201
5	Italy	29	565
6	Austria	27	843
7	France	26	401
8	Australia	26	278
9	Canada	26	268
10	South Korea	21	466

AI = artificial intelligence.

### 3.4. Co-authorship analysis

Co-authorship analysis quantifies the interconnections between authors, institutions, and countries by counting the number of papers that appear together. It is among the most crucial indicators for understanding the cooperation status and identifying prominent authors, institutions, and countries/regions.

VOS viewer generates visualization maps, where distinct nodes on the maps correspond to various authors, institutions, or countries, and the magnitude of the nodes is commensurate with the number of papers they represent. The interconnecting lines between the nodes signify cooperation among authors, institutions, or countries. The thickness of the lines, as determined by the link strength (LS), was proportional to the number of coauthored papers. The total link strength (TLS) is the sum of the total LS for a specific author, institution, or country.

**Figure 3.**
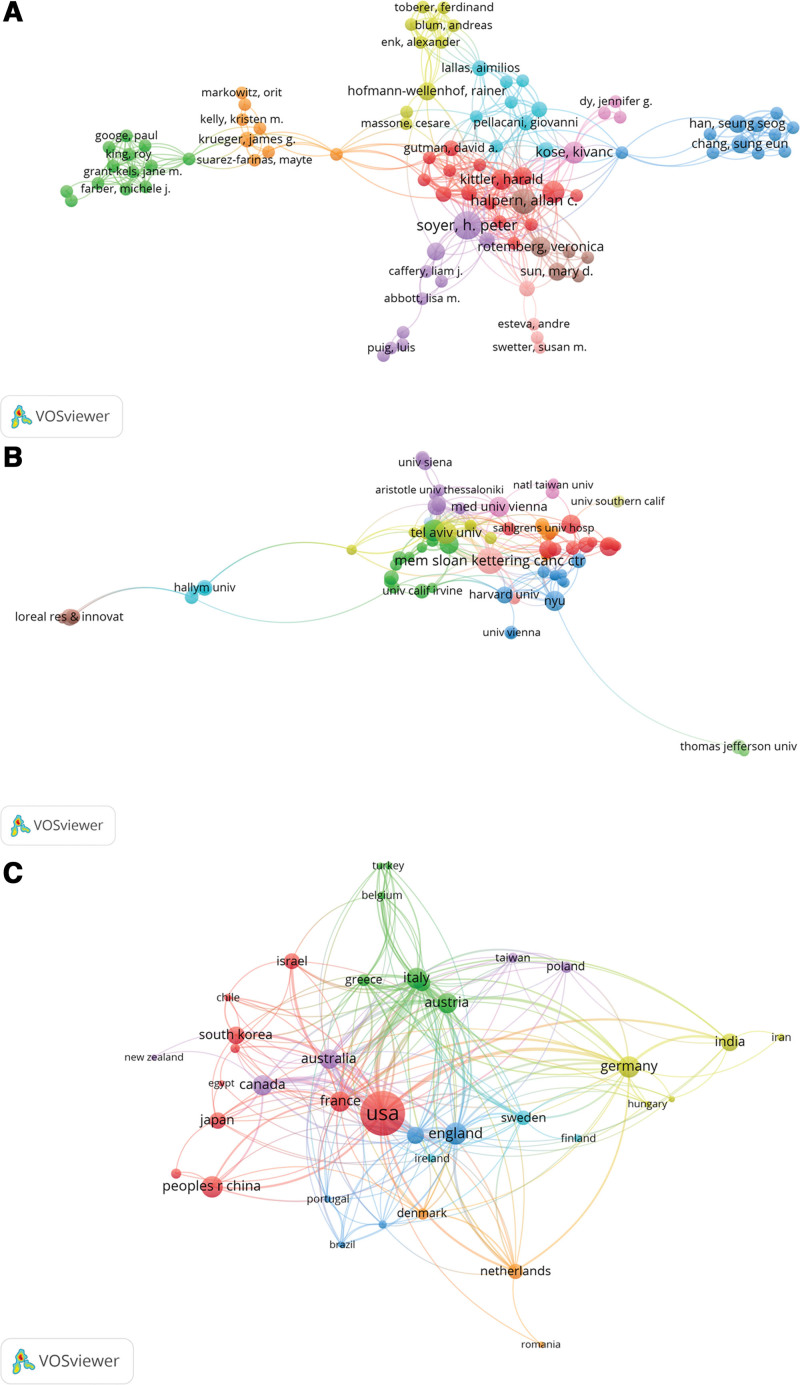
Visualization maps of co-authorship analysis using VOSviewer. Co-authorship analysis of (A) authors, (B) institutions, and (C) countries concerning AI applications in dermatology. The magnitude of the nodes pictured on the visualization maps is commensurate with the number of papers that they represent. The lines that interconnect nodes signify cooperative relationships among authors, institutions, or countries, and the thickness of the lines is directly proportional to the number of coauthored papers. AI = artificial intelligence.

A minimum of 2 papers and zero citations were set for an author to identify co-authorship. We selected 253 authors for the co-authorship analysis that met the stated thresholds. Figure 3A demonstrates that Frederic Flament had the strongest collaborative relationship (TLS = 48), followed by Ruowei Jiang (TLS = 47), H. Peter Soyer (TLS = 46), Allan C. Halpern (TLS = 42), and Yuze Zhang (TLS = 38). Allan C. Halpern and H. Peter Soyer had the strongest collaboration, followed by Allan C. Halpern and Mary D. Sun, H. Peter Soyer and Monika Janda, and Harald Kittler and Philipp Tschandl (LS = 3).

Similarly, we performed institutional co-authorship analysis. This assessment set a minimum of 3 papers and zero citations for each institution. We selected 87 institutions that met the thresholds for co-authorship analysis. Figure 3B shows that the Memorial Sloan Kettering Cancer Center had the strongest collaborative relationship (TLS = 53), followed by the University of Queensland (TLS = 36). The Memorial Sloan Kettering Cancer Center and New York University had the strongest collaboration (LS = 5), followed by the University of Queensland and the Medical University of Graz (LS = 4).

For countries/regions, a minimum of 2 papers and zero citations were set for each country. We selected 39 countries/regions that met the required thresholds for co-authorship analysis. The United States conducts the most international cooperative research (TLS = 107), followed by the United Kingdom (TLS = 70). Figure 3C illustrates that Canada and France had the highest levels of collaboration (LS = 11), followed by Australia and the United Kingdom (LS = 10).

### 3.5. Bibliometric analysis of keywords

**Figure 4.**
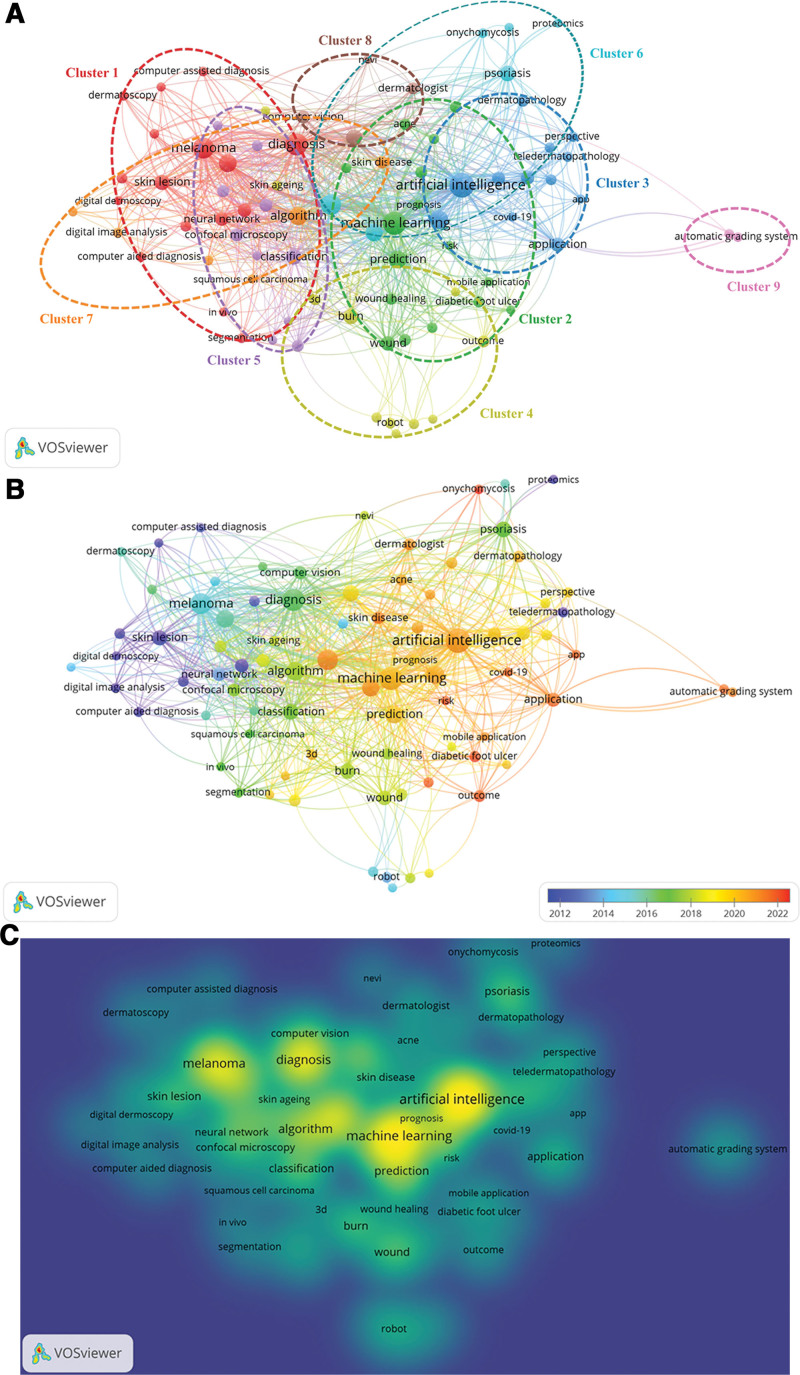
Visualization maps of keyword co-occurrence analysis using VOSviewer. (A) Network map. The keywords are categorized into nine distinct clusters. (B) Overlay map. The hues of the nodes serve as an indicator of the average publication year of the keyword co-occurrence. The blue nodes indicate that the keywords were present earlier, whilst the red nodes indicate that the keywords became relevant more recently. (C) Density map. The spatial arrangement of keywords is based on their frequency of co-occurrence, with the most frequent keywords highlighted in yellow, followed by those in green, cyan, and blue.

A keyword co-occurrence analysis quantitatively examines the fundamental attributes of keywords, including their co-occurrence frequency and evolution over time. We can understand the research status and development trends in certain fields by analyzing the co-occurring keywords. In this study, a minimum of 4 times was set for the occurrence of an author’s keywords. We identified 83 author keywords that met the predetermined thresholds mentioned above and then performed keyword co-occurrence analysis using VOSviewer. Figure 4 depicts 3 types of keyword visualization maps based on co-occurrence data: network, density, and overlay maps. The network map categorized the selected keywords into 9 clusters (Fig. 4A). Clusters 1 and 2 were the largest with 16 terms each. Table 5 lists the dominant keywords in each cluster. The overlay map shows changes in keywords over time (Fig. 4B). In this map, keywords are colored differently based on their average publication year, which is a term used to evaluate keyword novelty. The blue nodes indicate that the keywords were presented earlier, while the red nodes indicate that the keywords became relevant more recently. Early research on AI applications in dermatology primarily focused on “computer-assisted diagnosis of melanoma using artificial neural network”. Subsequently, keywords such as algorithm, burn, and confocal microscopy emerged. The keywords present in clusters 2, 3, and 7, namely onychomycosis, diabetic foot ulcer, risk, outcome, APP, and chronic wound, exhibited comparatively recent average publication years of 2021.80, 2021.67, 2021.67, 2021.56, 2021.50, 2021.50, and 2021.50, respectively, suggesting that they are receiving increasing attention and are likely to remain significant subjects of research. The density map illustrates the spatial arrangement of the keywords according to their co-occurrence frequencies. Notably, the keywords highlighted in yellow had the highest frequency, followed by those in green, cyan, and blue (Fig. 4C).

**Table 5:** Nine distinct clusters grouped by the analysis of keyword co-occurrence.

Clusters	Research hotspots	Number of items	Main keywords
Cluster 1	Computer-assisted diagnosis of melanoma using artificial neural network	16	Diagnosis, melanoma, artificial neural network
Cluster 2	Prediction of wound progression using machine learning	16	Machine learning, prediction, wound, diabetic foot ulcer
Cluster 3	Integration of AI into teledermatology	12	Artificial intelligence, application, telemedicine, teledermatology
Cluster 4	Utility of robots in burn and hair restoration	11	Burn, hair restoration, robot, robotic surgery
Cluster 5	Classification of skin disease via image analysis	10	Classification, image analysis, basal cell carcinoma
Cluster 6	Deep learning applications for Psoriasis	6	Psoriasis, deep learning
Cluster 7	Applications of algorithms in skin diseases	6	Algorithm, skin disease
Cluster 8	Computer vision system and Dermatologist for skin cancer	4	Computer vision, skin cancer, dermatologist
Cluster 9	Automatic grading system of facial signs	2	Automatic grading system, facial sign

AI = artificial intelligence.

**Figure 5.**
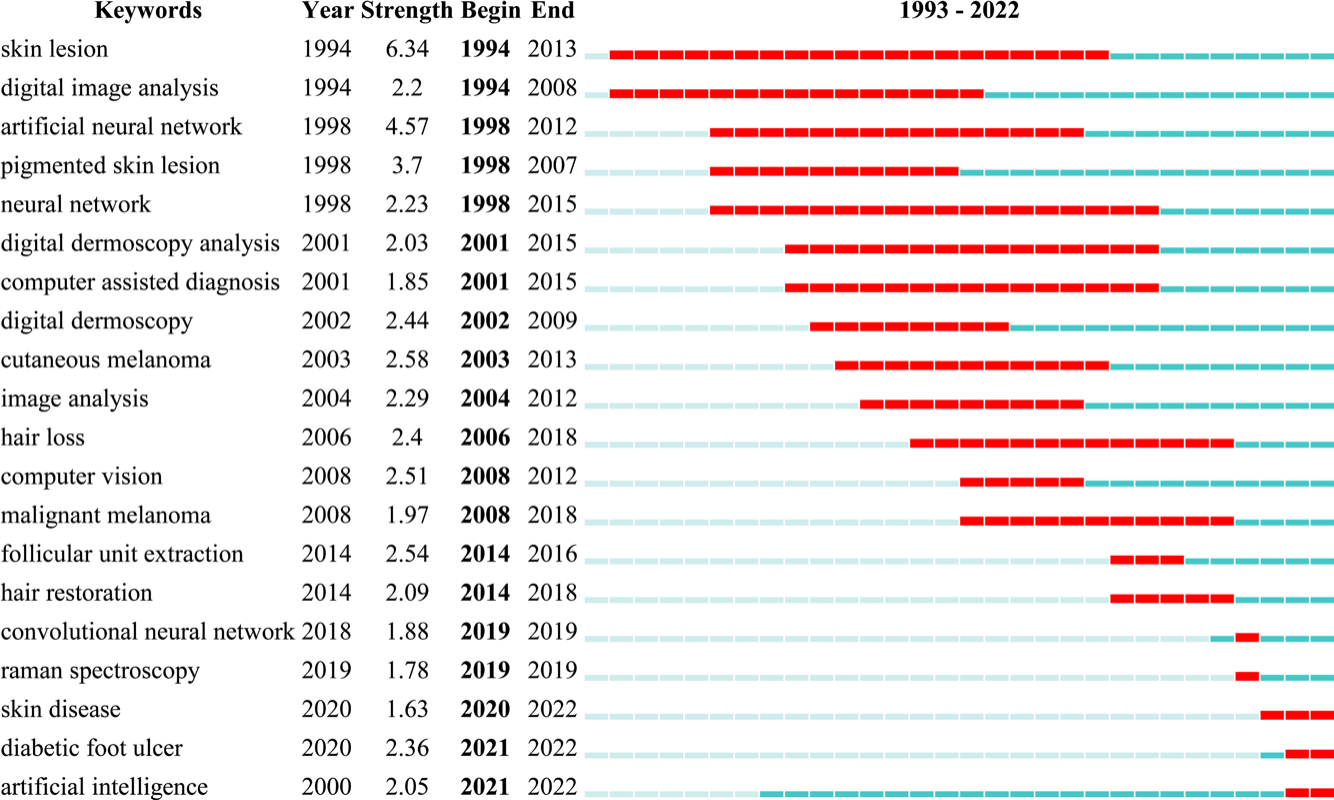
Top 20 keywords with the strongest citation bursts.

Additionally, we analyzed the progression of emerging keywords over time to better understand the applications of AI in dermatology. Figure 5 presents the top 20 keywords with the strongest citation bursts. The blue line represents the time interval, and the red line represents the outbreak duration. The keywords “skin lesion” (1994–2013), “neural network” (1998–2015), “digital image analysis” (1994–2008), “artificial neural network” (1998–2012), “digital dermoscopy analysis” (2001–2015), and “computer assisted diagnosis” (2001–2015) have received attention for the longest time in the past. The keywords “skin disease” (2020–2022), “diabetic foot ulcer” (2021–2022), and “artificial intelligence” (2021–2022) have been used more recently, and their usage continues.

The blue line represents the time interval and the red line represents the duration of the outbreak.

## 4. Discussion

### 4.1. General information

Microsoft Excel 2019, VOS viewer, and Cite Space were used to analyze 406 papers retrieved from the WoS Core Collection on AI applications in dermatology, thereby identifying knowledge hotspots and future research directions. We discovered that randomized controlled trials comprised 2% of all papers, whereas prospective studies comprised 5%, highlighting the need for further randomized controlled trials and prospective studies to validate the applications of AI in dermatology practice.

The annual number and evolving patterns of published papers indicate the pace and advancement of AI research in dermatology. The oldest paper in this field was published in 1985, and the number of papers released annually has steadily increased over the past 33 years. The turning point occurred in 2018, when the number of papers increased rapidly. This was likely due to advancements in AI technology, such as neural networks, autonomous robots, and machine learning, which offer unprecedented disease prediction, diagnosis, and management opportunities. The number of annual papers will continue to increase according to developmental trends.

Core journals can be identified by analyzing the source journals of papers related to AI research in dermatology. Our results indicated that the preferred journals for researchers were *Skin Research and Technology, Journal of the European Academy of Dermatology and Venereology*, and *Journal of the American Academy of Dermatology*. Seven of the top 10 most productive journals had Cite Scores > 5, indicating that research on AI applications in dermatology has received extensive attention.

Allan C. Halpern has published the largest number of papers in this field. Additionally, Halpern papers were the most frequently cited, suggesting that Halpern was the most influential scholar with a comprehensive and in-depth understanding of AI applications in dermatology^.[33,34]^ According to the country distribution of papers, the United States produced the most papers, accounting for 40.9% of the total papers on AI research in dermatology. This finding is consistent with the outcomes of bibliometric investigations of AI applications in diverse medical fields, including intensive care medicine.^[35]^ Papers from the United States received the highest number of citations, suggesting that the United States has emerged as the foremost nation for AI research in dermatology. This discovery can be ascribed to various factors, such as substantial financial investment, many researchers, a considerable proportion of foreign graduate students or visiting scholars, and vigorous engagement in international cooperation. European countries/regions, including the United Kingdom, Germany, Italy, Austria, France, and Switzerland published 269 papers. North American countries including the United States, Canada, and Mexico published 194 papers. Asian countries, including China, India, South Korea, Japan, and Iran published 132 papers. Oceanian countries, including Australia and New Zealand, published 28 papers. African countries, including South Africa and Benini, published 19 papers. South American countries published 7 papers.

Seven of the 10 most prolific institutions are affiliated with the United States, with one each from Australia, Israel, and Austria. The University of California Systems published the largest number of papers. This may have occurred because of various factors, including its ranking as the largest public university system in the United States and its great resources for productive research groups. Regarding institutional collaboration, the Memorial Sloan Kettering Cancer Center in the United States has the closest cooperative relationship with multiple institutions, including Australia and Austria.

### 4.2. Research hotspots and future directions

We conducted a co-occurrence analysis of keywords using VOS viewer to investigate the status of AI applications in dermatology. Based on the network map, we divided all selected keywords into 9 distinct clusters. Cluster 1 of the 9 distinct clusters represents one of the most prominent research areas of AI applications in dermatology, such as computer-assisted diagnosis of melanoma using artificial neural networks. As medicine enters a new era of AI-assisted medicine, AI is being integrated into dermatology for skin disease diagnosis, treatment, and prognostic prediction, particularly for melanoma diagnosis. Cascinelli et al^[36]^ developed a computerized image analysis system and concluded that it should be viewed as a useful diagnostic aid for nonexpert clinicians.

Cluster 2 revealed another research hotspot: the prediction of wound progression using machine learning. Machine learning accurately predicts the risk factors, clinical remission time, drug response, and wound prognosis in dermatology. Xu et al^[37]^ developed a scoring nomogram based on machine learning to facilitate the early detection of potential pressure injuries in an intensive care unit by utilizing extensive clinical data from 2022. Their research indicated that implementing machine learning techniques enhanced the predictive accuracy of routinely documented clinical data concerning pressure injury progress. Cluster 3 focused on the integration of AI into teledermatology. Teledermatology has made dermatologist care more accessible to patients. Patients or designated individuals can take photographs of skin diseases and transmit them to an AI platform for assessment, diagnosis, and treatment. This impact is felt in geographical areas traditionally associated with difficulties in accessing doctors and extended patient waiting times.^[38]^

Cluster 4 primarily involved the use of robots for hair restoration and burns. With the development of robotics, robotic surgery has been increasingly used in hair restoration. A retrospective study found that a robotic device designed for recipient site creation is a safe and dependable clinical tool. This device automatically produces slit incisions in the recipient area at a speed and consistency comparable to those created manually. Furthermore, it can easily be controlled by a diverse range of hair surgeons.^[18]^ Cluster 5 emphasized skin disease classification via image analysis. Several studies have analyzed clinical and dermatopathological images to categorize skin lesions associated with various skin conditions such as basal cell carcinoma, melanoma, atopic dermatitis, onychomycosis, and rosacea.^[39]^

Cluster 6 discussed deep learning applications for psoriasis. Nielsen et al^[40]^ developed treatment prediction models for patients with psoriasis and concluded that predicting the optimal biological therapy could reduce failed treatment attempts and benefit both patients and clinicians. Cluster 7 was related to algorithm applications for skin diseases. Timely and accurate diagnosis of infantile hemangiomas is important to prevent potential complications. Researchers trained an AI algorithm to diagnose infantile hemangiomas using clinical images in an article published in Pediatric Dermatology in 2022. This algorithm demonstrated a diagnostic accuracy of 91.7% for facial hemangiomas in infants.^[41]^

Cluster 8 primarily referred to computer vision systems and skin cancer dermatologists. Friedman RJ and colleagues conducted a blinded comparison study to evaluate the diagnostic performance of dermoscopic and automatic multispectral computer vision systems for detecting small, pigmented skin lesions. This study revealed that computer vision systems can detect small melanomas earlier, thereby reducing the number of biopsies performed on benign lesions.^[42]^ Focus shifted to an automatic facial sign grading system in cluster 9. Zhang et al^[43]^ demonstrated that an AI-based automated scoring system, designed specifically to detect certain facial features in men and unaffected by human factors, performed at the same level as assessments previously developed for women, and its results closely matched those of expert assessments.

We performed a keyword overlay and density mapping using VOS viewer to predict future research directions. Moreover, we conducted keyword burstiness analysis using Cite Space software to conduct an overlap analysis. The overlay map displays the temporal progression of keywords from the initial study focused on cluster 1, “computer-assisted diagnosis of melanoma using artificial neural network”, to cluster 5, entitled “classification of skin disease by image analysis”, and the current dimensions of cluster 2, “wound progression prediction using machine learning”; cluster 3, named “integration of AI into teledermatology” and cluster 7, the “applications of the algorithms in skin diseases”. The visualization map suggests that the latter 3 clusters may be future research hotspots. Density visualization exhibited several “marginal” keywords, such as diabetic foot ulcer, precision medicine, digital image analysis, and APP, in clusters 2, 3, and 7, respectively. Despite not being representative of the entire field, these keywords raise important questions and suggest that future research should be directed toward these areas. Additionally, citation burst detection for keywords highlighted that the terms “skin disease,” “diabetic foot ulcer,” and “AI” started to emerge in 2020, 2021, and 2021, respectively, and this trend has continued to the present. This suggests that these research hotspots have received consistent attention in recent years and may be future research directions for AI applications in dermatology. These 3 keywords were included in clusters 2, 3, and 7 of the network map produced using VOS viewer.

### 4.3. Research limitations

This study provides a comprehensive review of the current status and prospects of dermatology-related AI studies; identifies the most productive authors, countries, and institutions for possible collaborations; and predicts future research directions in this field. However, several limitations should be considered when interpreting the results. First, papers on AI applications in dermatology were retrieved from WoS Core Collection. However, studies from other databases, such as PubMed, Scopus, and Google Scholar, were excluded. Additionally, despite our efforts to incorporate a comprehensive range of AI-related terms into our search criteria, the possibility of retaining certain terms remains. Thus, the search results may not have covered all AI-related dermatology papers. Second, bibliometric metadata vary over time, indicating that different conclusions can be drawn in the future. Further updates are necessary to improve the accuracy of this study. Third, papers published in languages other than English were excluded. Thus, we might have overlooked relevant studies conducted in other languages. For example, researchers from China may regularly publish their work in Chinese journals, resulting in the underestimation of their contributions.

## 5. Conclusion

To our knowledge, this is the first bibliometric analysis of AI applications in dermatology. This study presents a comprehensive summary of the current research on AI in dermatology based on 406 papers retrieved from the WoS Core Collection database. The number of papers published on AI applications in dermatology has increased significantly over the last half-decade, with projections indicating a continued upward trend. We determined that the United States was the most influential nation during this period. Further randomized controlled trials and prospective studies are required to validate the applications of AI in dermatology. Wound progression prediction using machine learning, integration of AI into teledermatology, and algorithm applications in skin diseases, are current research priorities and will remain future research aims in this field.

## Acknowledgments

We would like to thank Editage (www.editage.com) and HOME for Researchers (www.home-for-researchers.com) for English language editing. This study was funded by the Science and Technology Project of Jinan, China (202019139).

## Author contributions

**Conceptualization:** Guangxin Wang.

**Data curation:** Guangxin Wang, Xianguang Meng.

**Formal analysis:** Guangxin Wang, Fan Zhang.

**Funding acquisition:** Guangxin Wang.

**Investigation:** Guangxin Wang, Xianguang Meng, Fan Zhang.

**Project administration:** Guangxin Wang, Xianguang Meng.

**Resources:** Fan Zhang.

**Supervision:** Guangxin Wang.

**Writing – original draft:** Guangxin Wang, Xianguang Meng.

**Writing – review & editing:** Guangxin Wang.

## Supplementary Material


