# Supplementary material for: Past, present, and future of global research on artificial intelligence applications in dermatology: A bibliometric analysis
Source: Medicine (Baltimore). 2023 Nov 10;102(45):e35993. doi: 10.1097/MD.0000000000035993 (PMC10637496; doi:10.1097/MD.0000000000035993)
Supplement: Supplementary file 1 [file medi-102-e35993-s001.docx]

Supplemental Digital Content

Past, present, and future of global research on artificial intelligence applications in dermatology: A bibliometric analysis

Guangxin Wang*, Xianguang Meng, Fan Zhang

*** Correspondence:** Guangxin Wang: wanggxchina@sdu.edu.cn

# Supplementary Data S1. The selected 406 papers regarding AI applications in dermatology.

1. Abbott LM, Smith SD. Smartphone apps for skin cancer diagnosis: Implications for patients and practitioners. Australasian Journal of Dermatology. 2018;59(3):168-70.
2. Adabi S, Fotouhi A, Xu QY, Daveluy S, Mehregan D, Podoleanu A, et al. An overview of methods to mitigate artifacts in optical coherence tomography imaging of the skin. Skin Research and Technology. 2018;24(2):265-73.
3. Aggarwal P. Data augmentation in dermatology image recognition using machine learning. Skin Research and Technology. 2019;25(6):815-20.
4. Aggarwal PI, Papay FA. Artificial intelligence image recognition of melanoma and basal cell carcinoma in racially diverse populations. Journal of Dermatological Treatment. 2022;33(4):2257-62.
5. Aghaei A, Soori H, Ramezankhani A, Mehrabi Y. Factors Related to Pediatric Unintentional Burns: The Comparison of Logistic Regression and Data Mining Algorithms. Journal of Burn Care & Research. 2019;40(5):606-12.
6. Al-Qarqaz F, Bodoor K, Baba A, Al-Yousef A, Muhaidat J, Alshiyab D. Post-acne hyperpigmentation: Evaluation of risk factors and the use of artificial neural network as a predictive classifier. Dermatology Reports. 2021;13(3):8223.
7. Ali S, Potokar TS, Chamania S, Lohana P, Price P, Whitaker IS. A novel, cost effective escharotomy simulator and trainee assessment. Burns. 2008;34(4):531-2.
8. Alkhushayni S, Al-zaleq D, Andradi L, Flynn P. The Application of Differing Machine Learning Algorithms and Their Related Performance in Detecting Skin Cancers and Melanomas. Journal of Skin Cancer. 2022;2022:2839162.
9. Amruthalingam L, Gottfrois P, Jimenez AG, Gokduman B, Kunz M, Koller T, et al. Improved diagnosis by automated macro- and micro-anatomical region mapping of skin photographs. Journal of the European Academy of Dermatology and Venereology. 2022;36(12):2525-32.
10. Andres C, Andres-Belloni B, Hein R, Biedermann T, Schape A, Brieu N, et al. iDermatoPath - a novel software tool for mitosis detection in H&E-stained tissue sections of malignant melanoma. Journal of the European Academy of Dermatology and Venereology. 2017;31(7):1137-47.
11. Anisuzzaman DM, Wang CB, Rostami B, Gopalakrishnan S, Niezgoda J, Yu ZY. Image-Based Artificial Intelligence in Wound Assessment: A Systematic Review. Advances in Wound Care. 2022;11(12):687-709.
12. Aractingi S, Pellacani G. Computational neural network in melanocytic lesions diagnosis: artificial intelligence to improve diagnosis in dermatology? European Journal of Dermatology. 2019;29:4-7.
13. Armstrong DG, Rowe VL, D'Huyvetter K, Sherman RA. Telehealth-guided home-basedmaggot debridement therapy for chronic complex wounds: Peri- andpost-pandemicpotential. International Wound Journal. 2020;17(5):1490-5.
14. Avram MR, Finney R, Rogers N. Hair Transplantation Controversies. Dermatologic Surgery. 2017;43:S158-S62.
15. Avram MR, Watkins SA. Robotic Follicular Unit Extraction in Hair Transplantation. Dermatologic Surgery. 2014;40(12):1319-27.
16. Baek KW, Dard M, Zeilhofer HF, Cattin PC, Juergens P. Comparing the Bone Healing After Cold Ablation Robot-Guided Er:YAG Laser Osteotomy and Piezoelectric Osteotomy-A Pilot Study in a Minipig Mandible. Lasers in Surgery and Medicine. 2021;53(3):291-9.
17. Baharestani M. Clinical decision-making in wound care management - the need for a paradigmatic shift. Wounds-a Compendium of Clinical Research and Practice. 1995;7:A84-A9.
18. Bao YY, Zhang JY, Zhao XY, Zhou HH, Chen Y, Jian JM, et al. Deep learning-based fully automated diagnosis of melanocytic lesions by using whole slide images. Journal of Dermatological Treatment. 2022;33(5):2571-7.
19. Barak-Levitt J, Held R, Synett Y, Kremer N, Hodak E, Sherman S. Hidradenitis Suppurativa International Online Community: Patient Characteristics and a Novel Model of Treatment Effectiveness. Acta Dermato-Venereologica. 2022;102: adv00686.
20. Barakat-Johnson M, Jones A, Burger M, Leong T, Frotjold A, Randall S, et al. Reshaping wound care: Evaluation of an artificial intelligence app to improve wound assessment and management amid the COVID-19 pandemic. International Wound Journal. 2022;19(6):1561-77.
21. Bargo PR, Kollias N. Measurement of skin texture through polarization imaging. British Journal of Dermatology. 2010;162(4):724-31.
22. Basu K, Sinha R, Ong A, Basu T. Artificial Intelligence: How is It Changing Medical Sciences and Its Future? Indian Journal of Dermatology. 2020;65(5):365-70.
23. Basu T, Engel-Wolf S, Menzer O. The Ethics of Machine Learning in Medical Sciences: Where Do We Stand Today? Indian Journal of Dermatology. 2020;65(5):358-64.
24. Berezo M, Budman J, Deutscher D, Hess CT, Smith K, Hayes D. Predicting Chronic Wound Healing Time Using Machine Learning. Advances in Wound Care. 2022;11(6):281-96.
25. Bernstein RM, Wolfeld MB. Robotic Follicular Unit Graft Selection. Dermatologic Surgery. 2016;42(6):710-4.
26. Betz-Stablein B, D'Alessandro B, Koh U, Plasmeijer E, Janda M, Menzies SW, et al. Reproducible Naevus Counts Using 3D Total Body Photography and Convolutional Neural Networks. Dermatology. 2022;238(1):4-11.
27. Binder M, Kittler H, Dreiseitl S, Ganster H, Wolff K, Pehamberger H. Computer-aided epiluminescence microscopy of pigmented skin lesions: the value of clinical data for the classification process. Melanoma Research. 2000;10(6):556-61.
28. Binder M, Kittler H, Seeber A, Steiner A, Pehamberger H, Wolff K. Epiluminescence microscopy-based classification of pigmented skin lesions using computerized image analysis and an artificial neural network. Melanoma Research. 1998;8(3):261-6.
29. Binder M, Steiner A, Schwarz M, Knollmayer S, Wolff K, Pehamberger H. Application of an artificial neural network in epiluminescence microscopy pattern analysis of pigmented skin lesions: A pilot study. British Journal of Dermatology. 1994;130(4):460-5.
30. Binol H, Plotner A, Sopkovich J, Kaffenberger B, Niazi MKK, Gurcan MN. Ros-NET: A deep convolutional neural network for automatic identification of rosacea lesions. Skin Research and Technology. 2020;26(3):413-21.
31. Bleicher B, Levine A, Markowitz O. Going Digital With Dermoscopy. Cutis. 2018;102(2):102-5.
32. Blum A, Luedtke H, Ellwanger U, Schwabe R, Rassner G, Garbe C. Digital image analysis for diagnosis of cutaneous melanoma. Development of a highly effective computer algorithm based on analysis of 837 melanocytic lesions. British Journal of Dermatology. 2004;151(5):1029-38.
33. Boldrick JC, Layton CJ, Nguyen J, Swetter SM. Evaluation of digital dermoscopy in a pigmented lesion clinic: Clinician versus computer assessment of malignancy risk. Journal of the American Academy of Dermatology. 2007;56(3):417-21.
34. Bonnekoh B, Bockelmann R, Pommer AJ, Malykh Y, Philipsen L, Gollnick H. The CD11a binding site of efalizumab in psoriatic skin tissue as analyzed by multi-epitope ligand cartography robot technology - Introduction of a novel biological drug-binding biochip assay. Skin Pharmacology and Physiology. 2007;20(2):96-111.
35. Bonnekoh B, Pommer AJ, Bockelmann R, Hofmeister H, Philipsen L, Gollnick H. Topo-proteomic in situ analysis of psoriatic plaque under efalizumab treatment. Skin Pharmacology and Physiology. 2007;20(5):237-52.
36. Braun RP, Gutkowicz-Krusin D, Rabinovitz H, Cognetta A, Hofmann-Wellenhof R, Ahlgrimm-Siess V, et al. Agreement of Dermatopathologists in the Evaluation of Clinically Difficult Melanocytic Lesions: How Golden Is the 'Gold Standard'? Dermatology. 2012;224(1):51-8.
37. Braun RP, Rabinovitz HS, Oliviero M, Kopf AW, Saurat JH. Dermoscopy of pigmented skin lesions. Journal of the American Academy of Dermatology. 2005;52(1):109-21.
38. Breugnot J, Rouaud-Tinguely P, Gilardeau S, Rondeau D, Bordes S, Aymard E, et al. Utilizing deep learning for dermal matrix quality assessment on in vivo line-field confocal optical coherence tomography images. Skin Research and Technology. 2023;29(1):e13221.
39. Caffery LJ, Janda M, Miller R, Abbott LM, Arnold C, Caccetta T, et al. Informing a position statement on the use of artificial intelligence in dermatology in Australia. Australasian Journal of Dermatology. 2023;64(1):E11-E20.
40. Campanella G, Navarrete-Dechent C, Liopyris K, Monnier J, Aleissa S, Minhas B, et al. Deep Learning for Basal Cell Carcinoma Detection for Reflectance Confocal Microscopy. Journal of Investigative Dermatology. 2022;142(1):97-103.
41. Cascinelli N, Ferrario M, Bufalino R, Zurrida S, Galimberti V, Mascheroni L, et al. Results obtained by using a computerized image analysis system designed as an aid to diagnosis of cutaneous melanoma. Melanoma Research. 1992;2(3):163-70.
42. Cazzaniga S, Sassi F, Mercuri SR, Naldi L. Prediction of Clinical Response to Excimer Laser Treatment in Vitiligo by Using Neural Network Models. Dermatology. 2009;219(2):133-7.
43. Cazzato G, Colagrande A, Cimmino A, Arezzo F, Loizzi V, Caporusso C, et al. Artificial Intelligence in Dermatopathology: New Insights and Perspectives. Dermatopathology. 2021;8(3):418-25.
44. Chan KS, Chan YM, Tan AHM, Liang SY, Cho YT, Hong QT, et al. Clinical validation of an artificial intelligence-enabled wound imaging mobile application in diabetic foot ulcers. International Wound Journal. 2022;19(1):114-24.
45. Chan KS, Lo ZJ. Wound assessment, imaging and monitoring systems in diabetic foot ulcers: A systematic review. International Wound Journal. 2020;17(6):1909-23.
46. Chan S, Reddy V, Myers B, Thibodeaux Q, Brownstone N, Liao W. Machine Learning in Dermatology: Current Applications, Opportunities, and Limitations. Dermatology and Therapy. 2020;10(3):365-86.
47. Chauhan J, Goyal P. BPBSAM: Body part-specific burn severity assessment model. Burns. 2020;46(6):1407-23.
48. Chauhan J, Goyal P. Convolution neural network for effective burn region segmentation of color images. Burns. 2021;47(4):854-62.
49. Cheng BB, Stanley RJ, Stoecker WV, Stricklin SM, Hinton KA, Nguyen TK, et al. Analysis of clinical and dermoscopic features for basal cell carcinoma neural network classification. Skin Research and Technology. 2013;19(1):E217-E22.
50. Cheng B, Stanley RJ, Stoecker WV, Hinton K. Automatic telangiectasia analysis in dermoscopy images using adaptive critic design. Skin Research and Technology. 2012;18(4):389-96.
51. Cheraghlou S, Sadda P, Agogo GO, Girardi M. A machine-learning modified CART algorithm informs Merkel cell carcinoma prognosis. Australasian Journal of Dermatology. 2021;62(3):323-30.
52. Cho CH, Lee E, Park G, Cho E, Kim N, Shin J, et al. Evaluation of facial skin age based on biophysical properties in vivo. Journal of Cosmetic Dermatology. 2022;21(8):3546-54.
53. Cho YS, Joo SY, Seo CH. Effect of robot-assisted gait training on the biomechanical properties of burn scars: a single-blind, randomized controlled trial. Burns & Trauma. 2022;10:tkac026.
54. Choi BK, Kim MS, Kim SH. Risk prediction models for the development of oral-mucosal pressure injuries in intubated patients in intensive care units: A prospective observational study. Journal of Tissue Viability. 2020;29(4):252-7.
55. Christensen JH, Soerensen MBT, Zhong LH, Chen S, Jensen MO. Pre-diagnostic digital imaging prediction model to discriminate between malignant melanoma and benign pigmented skin lesion. Skin Research and Technology. 2010;16(1):98-108.
56. Cirillo MD, Mirdell R, Sjoberg F, Pham TD. Time-Independent Prediction of Burn Depth Using Deep Convolutional Neural Networks. Journal of Burn Care & Research. 2019;40(6):857-63.
57. Cirillo MD, Mirdell R, Sjoberg F, Pham TD. Improving burn depth assessment for pediatric scalds by AI based on semantic segmentation of polarized light photography images. Burns. 2021;47(7):1586-93.
58. Clayton K, Vallejo A, Sirvent S, Davies J, Porter G, Reading IC, et al. Machine learning applied to atopic dermatitis transcriptome reveals distinct therapy-dependent modification of the keratinocyte immunophenotype. British Journal of Dermatology. 2021;184(5):913-22.
59. Coates SJ, Kvedar J, Granstein RD. Teledermatology: From historical perspective to emerging techniques of the modern era Part II: Emerging technologies in teledermatology, limitations and future directions. Journal of the American Academy of Dermatology. 2015;72(4):577-88.
60. Cook MK, Kaszycki MA, Richardson I, Taylor SL, Feldman SR. Initial validation of a new device for facial skin analysis. Journal of Dermatological Treatment. 2022;33(8):3150-3.
61. Cowen EW, Liu CW, Steinberg SM, Kang S, Vonderheid EC, Kwak HS, et al. Differentiation of tumour-stage mycosis fungoides, psoriasis vulgaris and normal controls in a pilot study using serum proteomic analysis. British Journal of Dermatology. 2007;157(5):946-53.
62. Cui XY, Wei R, Gong LX, Qi RQ, Zhao ZY, Chen HD, et al. Assessing the effectiveness of artificial intelligence methods for melanoma: A retrospective review. Journal of the American Academy of Dermatology. 2019;81(5):1176-80.
63. Cullell-Dalmau M, Otero-Vinas M, Manzo C. Research Techniques Made Simple: Deep Learning for the Classification of Dermatological Images. Journal of Investigative Dermatology. 2020;140(3):507-14.
64. Cunningham L, Ganier C, Ferguson F, White IR, Watt FM, McFadden J, et al. Gradient boosting approaches can outperform logistic regression for risk prediction in cutaneous allergy. Contact Dermatitis. 2022;86(3):165-74.
65. Dabas M, Schwartz D, Beeckman D, Gefen A. Application of Artificial Intelligence Methodologies to Chronic Wound Care and Management: A Scoping Review. Advances in Wound Care. 2023;12(4):205-40.
66. Dai F, Zhang DY, Su KH, Xin N. Burn Images Segmentation Based on Burn-GAN. Journal of Burn Care & Research. 2021;42(4):755-62.
67. Damiani G, Buja A, Grossi E, Rivera M, De Polo A, De Luca G, et al. Use of an Artificial Neural Network to Identify Patient Clusters in a Large Cohort of Patients with Melanoma by Simultaneous Analysis of Costs and Clinical Characteristics. Acta Dermato-Venereologica. 2020;100: adv00323.
68. Damiani G, Conic RRZ, Pigatto PDM, Carrera CG, Franchi C, Cattaneo A, et al. Predicting Secukinumab Fast-Responder Profile in Psoriatic Patients: Advanced Application of Artificial-Neural-Networks (ANNs). Journal of Drugs in Dermatology. 2020;19(12):1241-6.
69. Damiani G, Grossi E, Berti E, Conic RRZ, Radhakrishna U, Pacifico A, et al. Artificial neural networks allow response prediction in squamous cell carcinoma of the scalp treated with radiotherapy. Journal of the European Academy of Dermatology and Venereology. 2020;34(6):1369-73.
70. Daneshjou R, Barata C, Betz-Stablein B, Celebi ME, Codella N, Combalia M, et al. Checklist for Evaluation of Image-Based Artificial Intelligence Reports in Dermatology CLEAR Derm Consensus Guidelines From the International Skin Imaging Collaboration Artificial Intelligence Working Group. Jama Dermatology. 2022;158(1):90-6.
71. Daneshjou R, Smith MP, Sun MD, Rotemberg V, Zou J. Lack of Transparency and Potential Bias in Artificial Intelligence Data Sets and Algorithms A Scoping Review. Jama Dermatology. 2021;157(11):1362-9.
72. Daniels G, Tamburic S, Benini S, Randall J, Sanderson T, Savardi M. Artificial Intelligence in hair research: A proof-of-concept study on evaluating hair assembly features. International Journal of Cosmetic Science. 2021;43(4):405-18.
73. de Koning SGB, Weijtmans P, Karakullukcu MB, Shan CF, Baltussen EJM, Smit LA, et al. Toward Assessment of Resection Margins Using Hyperspectral Diffuse Reflection Imaging (400-1,700 nm) During Tongue Cancer Surgery. Lasers in Surgery and Medicine. 2020;52(6):496-502.
74. De A, Sarda A, Gupta S, Das S. Use of Artificial Intelligence in Dermatology. Indian Journal of Dermatology. 2020;65(5):352-7.
75. Decroos F, Springenberg S, Lang T, Papper M, Zapf A, Metze D, et al. A Deep Learning Approach for Histopathological Diagnosis of Onychomycosis: Not Inferior to Analogue Diagnosis by Histopathologists. Acta Dermato-Venereologica. 2021;101:adv00532.
76. Desbois A, Beguet F, Leclerc Y, Hernandez AEG, Gervais S, Perreault I, et al. Predictive Modeling for Personalized Three-Dimensional Burn Injury Assessments. Journal of Burn Care & Research. 2020;41(1):121-30.
77. Dick V, Sinz C, Mittlbock M, Kittler H, Tschandl P. Accuracy of Computer-Aided Diagnosis of Melanoma A Meta-analysis. Jama Dermatology. 2019;155(11):1291-9.
78. Ding YC, Dhawan G, Jones C, Ness T, Nichols E, Krasnogor N, et al. An open source pipeline for quantitative immunohistochemistry image analysis of inflammatory skin disease using artificial intelligence. Journal of the European Academy of Dermatology and Venereology. 2023;37(3):605-14.
79. Dissanayake B, Miyamoto K, Purwar A, Chye R, Matsubara A. New image analysis tool for facial pore characterization and assessment. Skin Research and Technology. 2019;25(5):631-8.
80. Divito SJ, Ferris LK. Advances and short comings in the early diagnosis of melanoma. Melanoma Research. 2010;20(6):450-8.
81. dos Santos V, Hardt C, Skrede S, Saccenti E. Systems and Precision Medicine in Necrotizing Soft Tissue Infections. In: NorrbyTeglund A, Svensson M, Skrede S, editors. Necrotizing Soft Tissue Infections: Clinical and Pathogenic Aspects. Advances in Experimental Medicine and Biology. 2020; 1294:187-207.
82. Dover JS, Geronemus R, Stern RS, Ohare D, Arndt KA. Dye-laser treatment of port-wine stains - comparison of the continuous-wave dye-laser with a robotized scanning device and the pulsed dye laser. Journal of the American Academy of Dermatology. 1995;32(2):237-40.
83. Dreiseitl S, Binder M, Hable K, Kittler H. Computer versus human diagnosis of melanoma: evaluation of the feasibility of an automated diagnostic system in a prospective clinical trial. Melanoma Research. 2009;19(3):180-4.
84. Drysdale E, Peng YW, Nguyen P, Baetz T, Hanna TP. A population-based study of the treatment effect of first-line ipilimumab for metastatic or unresectable melanoma. Melanoma Research. 2019;29(6):635-42.
85. Du CZ, Li YY, Xie PG, Zhang X, Deng B, Wang GX, et al. The amputation and mortality of inpatients with diabetic foot ulceration in the COVID-19 pandemic and postpandemic era: A machine learning study. International Wound Journal. 2022;19(6):1289-97.
86. Du-Harpur X, Watt FM, Luscombe NM, Lynch MD. What is AI? Applications of artificial intelligence to dermatology. British Journal of Dermatology. 2020;183(3):423-30.
87. Dulmage B, Tegtmeyer K, Zhang MZ, Colavincenzo M, Xu S. A Point-of-Care, Real-Time Artificial Intelligence System to Support Clinician of a Wide of Skin Diseases. Journal of Investigative Dermatology. 2020;141(5):1230-5.
88. Eapen BR. Artificial intelligence in dermatology: A practical introduction to a paradigm shift. Indian Dermatology Online Journal. 2020;11(6):881-9.
89. Eibl-Lindner K, Furweger C, Nentwich M, Foerster P, Wowra B, Schaller U, et al. Robotic radiosurgery for the treatment of medium and large uveal melanoma. Melanoma Research. 2016;26(1):51-7.
90. Elder A, Ring C, Heitmiller K, Gabriel Z, Saedi N. The role of artificial intelligence in cosmetic dermatology-Current, upcoming, and future trends. Journal of Cosmetic Dermatology. 2021;20(1):48-52.
91. Engel TN, Abraham TM, Morningstar T, Fung MA, Rangchi A, Kiuru M, et al. Pilot study of fluorescence imitating brightfield imaging for rapid, slide-free dermatopathology. Journal of Cutaneous Pathology. 2022;49(12):1060-6.
92. Estahbanati HK, Bouduhi N. Role of artificial neural networks in prediction of survival of burn patients - a new approach. Burns. 2002;28(6):579-86.
93. Faita F, Oranges T, Di Lascio N, Ciompi F, Vitali S, Aringhieri G, et al. Ultra-high-frequency ultrasound and machine learning approaches for the differential diagnosis of melanocytic lesions. Experimental Dermatology. 2022;31(1):94-8.
94. Fajarnes GP, Santonja MM, Garcia BD, Lengua IL. Segmentation methods for acne vulgaris images: Proposal of a new methodology applied to fluorescence images. Skin Research and Technology. 2020;26(5):734-9.
95. Feizpour A, Marstrand T, Bastholm L, Eirefelt S, Evans CL. Label-Free Quantification of Pharmacokinetics in Skin with Stimulated Raman Scattering Microscopy and Deep Learning. Journal of Investigative Dermatology. 2021;141(2):395-403.
96. Felmingham CM, Adler NR, Ge ZY, Morton RL, Janda M, Mar VJ. The Importance of Incorporating Human Factors in the Design and Implementation of Artificial Intelligence for Skin Cancer Diagnosis in the Real World. American Journal of Clinical Dermatology. 2021;22(2):233-42.
97. Ferris LK, Harkes JA, Gilbert B, Winger DG, Golubets K, Akilov O, et al. Computer-aided classification of melanocytic lesions using dermoscopic images. Journal of the American Academy of Dermatology. 2015;73(5):769-76.
98. Fiala T, Lavin P. Safety of a 1064-nm robotic laser system for noninvasive lipolysis of the flanks. Lasers in Surgery and Medicine. 2022;54(5):672-81.
99. Filipescu SG, Butacu AI, Tiplica GS, Nastac DI. Deep-learning approach in the study of skin lesions. Skin Research and Technology. 2021;27(5):931-9.
100. Fink C, Blum A, Buhl T, Mitteldorf C, Hofmann-Wellenhof R, Deinlein T, et al. Diagnostic performance of a deep learning convolutional neural network in the differentiation of combined naevi and melanomas. Journal of the European Academy of Dermatology and Venereology. 2020;34(6):1355-61.
101. Finlay AY, Hammond P. expert systems in dermatology - the computer-potential - The example of facial tumor-diagnosis. Dermatologica. 1986;173(2):79-84.
102. Flament F, Jacquet L, Ye C, Amar D, Kerob D, Jiang R, et al. Artificial Intelligence analysis of over half a million European and Chinese women reveals striking differences in the facial skin ageing process. Journal of the European Academy of Dermatology and Venereology. 2022;36(7):1136-42.
103. Flament F, Jiang RW, Houghton J, Zhang YZ, Kroely C, Jablonski NG, et al. Accuracy and clinical relevance of an automated, algorithm-based analysis of facial signs from selfie images of women in the United States of various ages, ancestries and phototypes: A cross-sectional observational study. Journal of the European Academy of Dermatology and Venereology. 2023;37(1):176-83.
104. Flament F, Lee YW, Lee DH, Passeron T, Zhang YZ, Jiang RW, et al. The continuous development of a complete and objective automatic grading system of facial signs from selfie pictures: Asian validation study and application to women of three ethnic origins, differently aged. Skin Research and Technology. 2021;27(2):183-90.
105. Flament F, Maudet A, Ye CD, Zhang YZ, Jiang RW, Dubosc S, et al. Comparing the self-perceived effects of a facial anti-aging product to those automatically detected from selfie images of Chinese women of different ages and cities. Skin Research and Technology. 2021;27(5):880-90.
106. Flament F, Velleman D, Yamashita E, Nicolas A, Yokoyama E, Chibout S, et al. A 5-hour follow-up of the impact on ageing facial signs of some foundations in Japanese women through automatically analysed selfie pictures. International Journal of Cosmetic Science. 2022;44(4):431-9.
107. Flament F, Velleman D, Yamashita E, Nicolas A, Yokoyama E, Itaya A, et al. Japanese experiment of a complete and objective automatic grading system of facial signs from selfie pictures: Validation with dermatologists and characterization of changes due to age and sun exposures. Skin Research and Technology. 2021;27(4):544-53.
108. Flament F, Zhang YZ, Jiang RW, Trehin C, Cassier M, Delaunay C, et al. Objective and automatic grading system of facial signs from selfie pictures of South African women: Characterization of changes with age and sun-exposures. Skin Research and Technology. 2022;28(4):596-603.
109. Flament F, Zhang YZ, Yu Z, Jiang RW, Houghton J, Duthil LS, et al. Developing an Artificial Intelligence (A.I)-based descriptor of facial appearance that fits with the assessments of makeup experts. Skin Research and Technology. 2021;27(6):1081-91.
110. Fleming MG. Digital dermoscopy. Dermatologic Clinics. 2001;19(2):359-367.
111. Fong KY, Lai TP, Chan KS, Le See IJ, Goh CC, Muthuveerappa S, et al. Clinical validation of a smartphone application for automated wound measurement in patients with venous leg ulcers. International Wound Journal. 2023;20(3):751-60.
112. Fried L, Tan A, Bajaj S, Liebman TN, Polsky D, Stein JA. Technological advances for the detection of melanoma. Journal of the American Academy of Dermatology. 2020;83(4):983-95.
113. Friedman RJ, Farber MJ, Warycha MA, Papathasis N, Miller MK, Heilman ER. The "dysplastic" nevus. Clinics in Dermatology. 2009;27(1):103-15.
114. Friedman RJ, Gutkowicz-Krusin D, Farber MJ, Warycha M, Schneider-Kels L, Papastathis N, et al. The diagnostic performance of expert dermoscopists vs a computer-vision system on small-diameter melanomas. Archives of Dermatology. 2008;144(4):476-82.
115. Fujisawa Y, Otomo Y, Ogata Y, Nakamura Y, Fujita R, Ishitsuka Y, et al. Deep-learning-based, computer-aided classifier developed with a small dataset of clinical images surpasses board-certified dermatologists in skin tumour diagnosis. British Journal of Dermatology. 2019;180(2):373-81.
116. Galliano A, Guerin M, Lambert V, Favrot I, Seneca D, Lequeux F, et al. Virtual approach of the aesthetical fit between hair colours and skin tones in women of different ethnical origin backgrounds. Skin Research and Technology. 2022;28(3):455-64.
117. Gao A, Kouznetsova VL, Tsigelny IF. Machine-learning-based virtual screening to repurpose drugs for treatment of Candida albicans infection. Mycoses. 2022;65(8):794-805.
118. Gao M, Wang Y, Xu HP, Xu CC, Yang XH, Nie J, et al. Deep Learning-based Trichoscopic Image Analysis and Quantitative Model for Predicting Basic and Specific Classification in Male Androgenetic Alopecia. Acta Dermato-Venereologica. 2022;102:adv00635.
119. Gao WC, Li MR, Wu R, Du WA, Zhang SL, Yin SC, et al. The design and application of an automated microscope developed based on deep learning for fungal detection in dermatology. Mycoses. 2021;64(3):245-51.
120. Garbe C, Elgentler TK. Diagnosis and treatment of cutaneous melanoma: state of the art 2006. Melanoma Research. 2007;17(2):117-27.
121. Garcia P, Mines MJ, Bower KS, Hill J, Menon J, Tremblay E, et al. Robotic Laser Tissue Welding of Sclera Using Chitosan Films. Lasers in Surgery and Medicine. 2009;41(1):60-7.
122. Gareau DS, da Rosa JC, Yagerman S, Carucci JA, Gulati N, Hueto F, et al. Digital imaging biomarkers feed machine learning for melanoma screening. Experimental Dermatology. 2017;26(7):615-8.
123. Ge L, Li YY, Wu YG, Fan ZW, Song ZQ. Differential Diagnosis of Rosacea Using Machine Learning and Dermoscopy. Clinical Cosmetic and Investigational Dermatology. 2022;15:1465-73.
124. Gerger A, Pompl R, Smolle J. Automated epiluminescence microscopy - tissue counter analysis using CART and 1-NN in the diagnosis of melanoma. Skin Research and Technology. 2003;9(2):105-10.
125. Gerger A, Wiltgen M, Langsenlehner U, Richtig E, Horn M, Weger W, et al. Diagnostic image analysis of malignant melanoma in in vivo confocal laser-scanning microscopy: a preliminary study. Skin Research and Technology. 2008;14(3):359-63.
126. Gevaux L, Gierschendorf J, Rengot J, Cherel M, Seroul P, Nkengne A, et al. Real-time skin chromophore estimation from hyperspectral images using a neural network. Skin Research and Technology. 2021;27(2):163-77.
127. Gillstedt M, Mannius L, Paoli J, Gyllencreutz JD, Fougelberg J, Backman EJ, et al. Evaluation of Melanoma Thickness with Clinical Close-up and Dermoscopic Images Using a Convolutional Neural Network. Acta Dermato-Venereologica. 2022;102:adv00790.
128. Gillstedt M, Polesie S. Ability to Predict Melanoma Within 5 Years Using Registry Data and a Convolutional Neural Network: A Proof of Concept Study. Acta Dermato-Venereologica. 2022;102:adv00750.
129. Gilmore S, Hofmann-Wellenhof R, Soyer HP. A support vector machine for decision support in melanoma recognition. Experimental Dermatology. 2010;19(9):830-5.
130. Giuste F, Vizcarra JC, Gutman DA. Digital imaging applications and informatics in dermatology. Seminars in Cutaneous Medicine and Surgery. 2019;38(1):E43-E8.
131. Goldust Y, Sameem F, Mearaj S, Gupta A, Patil A, Goldust M. COVID-19 and artificial intelligence: Experts and dermatologists perspective. Journal of Cosmetic Dermatology. 2023;22(1):11-5.
132. Gooptu C, James MP. Recalcitrant viral warts: results of treatment with the KTP laser. Clinical and Experimental Dermatology. 1999;24(2):60-3.
133. Gupta AK, Hall DC. Diagnosing onychomycosis: A step forward? Journal of Cosmetic Dermatology. 2022;21(2):530-5.
134. Gupta AK, Ivanova IA, Renaud HJ. How good is artificial intelligence (AI) at solving hairy problems? A review of AI applications in hair restoration and hair disorders. Dermatologic Therapy. 2021;34(2):e14811.
135. Haberman HF, Norwich KH, Diehl DL, Evans SJ, Harvey B, Landau J, et al. DIAG - a computer-assisted dermatologic diagnostic system - clinical-experie and insight. Journal of the American Academy of Dermatology. 1985;12(1):132-43.
136. Hadeler E, Hong JL, Mosca M, Hakimi M, Brownstone N, Bhutani T, et al. Perspectives on the Future Development of Mobile Applications for Dermatology Clinical Research. Dermatology and Therapy. 2021;11(5):1451-6.
137. Hames SC, Bradley AP, Ardigo M, Soyer HP, Prow TW. Towards data-driven quantification of skin ageing using reflectance confocal microscopy. International Journal of Cosmetic Science. 2021;43(4):466-73.
138. Han SS, Kim MS, Lim W, Park GH, Park I, Chang SE. Classification of the Clinical Images for Benign and Malignant Cutaneous Tumors Using a Deep Learning Algorithm. Journal of Investigative Dermatology. 2018;138(7):1529-38.
139. Han SS, Kim YJ, Moon IJ, Jung JM, Lee MY, Lee WJ, et al. Evaluation of Artificial Intelligence-Assisted Diagnosis of Skin Neoplasms: A Single-Center, Paralleled, Unmasked, Randomized Controlled Trial. Journal of Investigative Dermatology. 2022;142(9):2353-62.
140. Han SS, Moon IJ, Lim W, Suh IS, Lee SY, Na JI, et al. Keratinocytic Skin Cancer Detection on the Face Using Region-Based Convolutional Neural Network. Jama Dermatology. 2020;156(1):29-37.
141. Han SS, Park I, Chang SE, Lim W, Kim MS, Park GH, et al. Augmented Intelligence Dermatology: Deep Neural Networks Empower Medical Professionals in Diagnosing Skin Cancer and Predicting Treatment Options for 134 Skin Disorders. Journal of Investigative Dermatology. 2020;140(9):1753-61.
142. Havelin A, Hampton P. Telemedicine and e-Health in the Management of Psoriasis: Improving Patient Outcomes - A Narrative Review. Psoriasis-Targets and Therapy. 2022;12:15-24.
143. Hejna M, Moon WM, Cheng J, Kawakami A, Fisher DE, Song JS. Local genomic features predict the distinct and overlapping binding patterns of the bHLH-Zip family oncoproteins MITF and MYC-MAX. Pigment Cell & Melanoma Research. 2019;32(4):500-9.
144. Hirano G, Nemoto M, Kimura Y, Kiyohara Y, Koga H, Yamazaki N, et al. Automatic diagnosis of melanoma using hyperspectral data and GoogLeNet. Skin Research and Technology. 2020;26(6):891-7.
145. Hoffmann K, Gambichler T, Rick A, Kreutz M, Anschuetz M, Grunendick T, et al. Diagnostic and neural analysis of skin cancer (DANAOS). A multicentre study for collection and computer-aided analysis of data from pigmented skin lesions using digital dermoscopy. British Journal of Dermatology. 2003;149(4):801-9.
146. Hogarty DT, Su JC, Phan K, Attia M, Hossny M, Nahavandi S, et al. Artificial Intelligence in Dermatology-Where We Are and the Way to the Future: A Review. American Journal of Clinical Dermatology. 2020;21(1):41-7.
147. Holm JG, Hurault G, Agner T, Clausen ML, Kezic S, Tanaka RJ, et al. Immunoinflammatory Biomarkers in Serum Are Associated with Disease Severity in Atopic Dermatitis. Dermatology. 2021;237(4):513-20.
148. Horsham C, Janda M, Kerr M, Soyer HP, Caffery LJ. Consumer perceptions on privacy and confidentiality in dermatology for 3D total-body imaging. Australasian Journal of Dermatology. 2023;64(1):118-21.
149. Hosking AM, Coakley BJ, Chang D, Talebi-Liasi F, Lish S, Lee SW, et al. Hyperspectral imaging in automated digital dermoscopy screening for melanoma. Lasers in Surgery and Medicine. 2019;51(3):214-22.
150. Howard J, Arango P, Ossoff J, Ossoff RH, Reinisch L. Healing of laser incisions in rat dermis: Comparisons of the carbon dioxide laser under manual and computer control and the scalpel. Lasers in Surgery and Medicine. 1997;20(1):90-6.
151. Howard T, Ahluwalia R, Papanas N. The Advent of Artificial Intelligence in Diabetic Foot Medicine: A New Horizon, a New Order, or a False Dawn? International Journal of Lower Extremity Wounds. 2021:1-6.
152. Huang HW, Hsu BWY, Lee CH, Tseng VS. Development of a light-weight deep learning model for cloud applications and remote diagnosis of skin cancers. Journal of Dermatology. 2021;48(3):310-6.
153. Huang KP, Mullangi S, Guo Y, Qureshi AA. Autoimmune, Atopic, and Mental Health Comorbid Conditions Associated With Alopecia Areata in the United States. Jama Dermatology. 2013;149(7):789-94.
154. Huang S, Dang J, Sheckter CC, Yenikomshian HA, Gillenwater J. A systematic review of machine learning and automation in burn wound evaluation: A promising but developing frontier. Burns. 2021;47(8):1691-704.
155. Hui HZ, Guo HX, Wang YJ, Shi BJ. Rare Case of Paraneoplastic Pemphigus Associated With Prostatic Cancer. American Journal of Dermatopathology. 2022;44(12):940-2.
156. Hung PK, Chu TW, Tsai RY, Kung CW, Lin SJ, Chen CM. Quantitative assessment of female pattern hair loss. Dermatologica Sinica. 2015;33(3):142-5.
157. Ibrahim SF, Taft BJ, Wang YP, Lee BI, Andrade E, Abaya C, et al. Minimally Invasive Skin Transcriptome Extraction Using a Dermal Biomarker Patch. Dermatology and Therapy. 2022;12(6):1313-23.
158. Jartarkar SR, Cockerell CJ, Patil A, Kassir M, Babaei M, Weidenthaler-Barth B, et al. Artificial intelligence in Dermatopathology. Journal of Cosmetic Dermatology. 2023;22(4):1163-7.
159. Jartarkar SR, Patil A, Wollina U, Gold MH, Stege H, Grabbe S, et al. New diagnostic and imaging technologies in dermatology. Journal of Cosmetic Dermatology. 2021;20(12):3782-7.
160. Jartarkar S, Patil A, Waskiel-Burnat A, Rudnicka L, Starace M, Grabbe S, et al. Artificial Intelligence in Hair and Nail Disorders. Journal of Drugs in Dermatology. 2022;21(10):1049-52.
161. Jiang YQ, Xiong JH, Li HY, Yang XH, Yu WT, Gao M, et al. Recognizing basal cell carcinoma on smartphone-captured digital histopathology images with a deep neural network. British Journal of Dermatology. 2020;182(3):754-62.
162. Jiao C, Su KH, Xie WG, Ye ZQ. Burn image segmentation based on Mask Regions with Convolutional Neural Network deep learning framework: more accurate and more convenient. Burns & Trauma. 2019;7(6):1-14.
163. Jobson D, Mar V, Freckelton I. Legal and ethical considerations of artificial intelligence in skin cancer diagnosis. Australasian Journal of Dermatology. 2022;63(1):E1-E5.
164. Jones JD, Rodriguez MR, Quinn KP. Automated Extraction of Skin Wound Healing Biomarkers From In Vivo Label-Free Multiphoton Microscopy Using Convolutional Neural Networks. Lasers in Surgery and Medicine. 2021;53(8):1086-95.
165. Jones OT, Jurascheck LC, Utukuri M, Pannebakker MM, Emery J, Walter FM. Dermoscopy use in UK primary care: a survey of GPs with a special interest in dermatology. Journal of the European Academy of Dermatology and Venereology. 2019;33(9):1706-12.
166. Joo SY, Lee SY, Cho YS, Yi SH, Seo CH. Clinical Utility of an Exoskeleton Robot Using Three-Dimensional Scanner Modeling in Burn Patient: A Case Report. Journal of Burn Care & Research. 2021;42(5):1030-4.
167. Jorgensen TM, Tycho A, Mogensen M, Bjerring P, Jemec GBE. Machine-learning classification of non-melanoma skin cancers from image features obtained by optical coherence tomography. Skin Research and Technology. 2008;14(3):364-9.
168. Jung K, Covington S, Sen CK, Januszyk M, Kirsner RS, Gurtner GC, et al. Rapid identification of slow healing wounds. Wound Repair and Regeneration. 2016;24(1):181-8.
169. Kaliyadan F, Manoj J, Dharmaratnam AD, Sreekanth G. Self-learning digital modules in Dermatology: a pilot study. Journal of the European Academy of Dermatology and Venereology. 2010;24(6):655-60.
170. Kanayama K, Kato H, Mori M, Sakae Y, Okazaki M. Robotically Assisted Recipient Site Preparation in Hair Restoration Surgery: Surgical Safety and Clinical Outcomes in 31 Consecutive Patients. Dermatologic Surgery. 2021;47(10):1365-70.
171. Karabulut YY, Dinc U, Kose EC, Tursen U. Deep learning as a new tool in the diagnosis of mycosis fungoides. Archives of Dermatological Research. 2023;315(5):1315-22.
172. Katz I, Azzi T, Lilleyman A, O'Brien B, Schapiro B, Thompson C, et al. Variability in the Histopathological Diagnosis of Non-Melanocytic Lesions Excised to Exclude Melanoma. Dermatology Practical & Conceptual. 2021;11(4):e2021094.
173. Keaney T. Emerging Therapies for Androgenetic Alopecia. Journal of Drugs in Dermatology. 2015;14(9):1036-40.
174. Kefel S, Guvenc P, LeAnder R, Stricklin SM, Stoecker WV. Discrimination of basal cell carcinoma from benign lesions based on extraction of ulcer features in polarized-light dermoscopy images. Skin Research and Technology. 2012;18(4):471-5.
175. Kenhagho HN, Canbaz F, Alvarez-Arenas TEG, Guzman R, Cattin P, Zam A. Machine Learning-Based Optoacoustic Tissue Classification Method for Laser Osteotomes Using an Air-Coupled Transducer. Lasers in Surgery and Medicine. 2021;53(3):377-89.
176. Kenhagho HN, Canbaz F, Hopf A, Guzman R, Cattin P, Zam A. Toward optoacoustic sciatic nerve detection using an all-fiber interferometric-based sensor for endoscopic smart laser surgery. Lasers in Surgery and Medicine. 2022;54(2):289-304.
177. Kennedy K, Cal R, Casey R, Lopez C, Adelfio A, Molloy B, et al. The anti-ageing effects of a natural peptide discovered by artificial intelligence. International Journal of Cosmetic Science. 2020;42(4):388-98.
178. Kertys M, Grendar M, Horak V, Zidekova N, Skalnikova HK, Mokry J, et al. Metabolomic characterisation of progression and spontaneous regression of melanoma in the melanoma-bearing Libechov minipig model. Melanoma Research. 2021;31(2):140-51.
179. Khodadad I, Shafiee J, Wong A, Kazemzadeh F, Arlette J. Deep Tissue Sequencing Using Hypodermoscopy and Augmented Intelligence to Analyze Atypical Pigmented Lesions. Journal of Cutaneous Medicine and Surgery. 2018;22(6):583-90.
180. Kim D, Ko M, Kim K. Skin tactile surface restoration using deep learning from a mobile image: An application for virtual skincare. Skin Research and Technology. 2021;27(5):739-50.
181. Kim RH, Nomikou S, Coudray N, Jour G, Dawood Z, Hong RY, et al. Deep Learning and Pathomics Analyses Reveal Cell Nuclei as Important Features for Mutation Prediction of BRAF-Mutated Melanomas. Journal of Investigative Dermatology. 2022;142(6):1650-8.
182. Kislal EE, Halasz CL. Software for quantifying psoriasis and vitiligo from digital clinical photographs. Journal of Dermatological Treatment. 2013;24(2):107-11.
183. Ko M, Kim D, Kim K. Accurate depth estimation of skin surface using a light-field camera toward dynamic haptic palpation. Skin Research and Technology. 2019;25(4):469-81.
184. Koller S, Wiltgen M, Ahlgrimm-Siess V, Weger W, Hofmann-Wellenhof R, Richtig E, et al. In vivo reflectance confocal microscopy: automated diagnostic image analysis of melanocytic skin tumours. Journal of the European Academy of Dermatology and Venereology. 2011;25(5):554-8.
185. Kose K, Bozkurt A, Alessi-Fox C, Brooks DH, Dy JG, Rajadhyaksha M, et al. Utilizing Machine Learning for Image Quality Assessment for Reflectance Confocal Microscopy. Journal of Investigative Dermatology. 2020;140(6):1214-22.
186. Krammer S, Li Y, Jakob N, Boehm AS, Wolff H, Tang P, et al. Deep learning-based classification of dermatological lesions given a limited amount of labelled data. Journal of the European Academy of Dermatology and Venereology. 2022;36(12):2516-24.
187. Kurugol S, Kose K, Park B, Dy JG, Brooks DH, Rajadhyaksha M. Automated Delineation of Dermal-Epidermal Junction in Reflectance Confocal Microscopy Image Stacks of Human Skin. Journal of Investigative Dermatology. 2015;135(3):710-7.
188. Kutzner H, Jutzi TB, Krahl D, Krieghoff-Henning EI, Heppt MV, Hekler A, et al. Overdiagnosis of melanoma - causes, consequences and solutions. Journal Der Deutschen Dermatologischen Gesellschaft. 2020;18(11):1236-43.
189. Kwiatkowska D, Kluska P, Reich A. Convolutional neural networks for the detection of malignant melanoma in dermoscopy images. Postepy Dermatologii I Alergologii. 2021;38(3):412-20.
190. Lallas A, Lallas K, Tschandl P, Kittler H, Apalla Z, Longo C, et al. The dermoscopic inverse approach significantly improves the accuracy of human readers for lentigo maligna diagnosis. Journal of the American Academy of Dermatology. 2021;84(2):381-9.
191. Landau M, Matz H, Tur E, Dvir M, Brenner S. Computerized system to enhance the clinical diagnosis of pigmented cutaneous malignancies. International Journal of Dermatology. 1999;38(6):443-6.
192. Laverde-Saad A, Jfri A, Garcia R, Salguero I, Martinez C, Cembrero H, et al. Discriminative deep learning based benignity/malignancy diagnosis of dermatologic ultrasound skin lesions with pretrained artificial intelligence architecture. Skin Research and Technology. 2022;28(1):35-9.
193. Lebwohl M, Soliman AM, Yang HB, Wang J, Freimark J, Puig L. Impact of PASI response on work productivity and the effect of risankizumab on indirect costs using machine learning in patients with moderate-to-severe psoriasis. Journal of Dermatological Treatment. 2022;33(4):2094-101.
194. Lee H, Ramani LT, Parish LC, Lee JB. Tools of dermatology: A historical perspective. Clinics in Dermatology. 2021;39(4):555-62.
195. Leem S, Gu KN, Kim Y, Jeong E, Lim JM, Kang NG. Two newly developed indices evaluating facial skin aging. Journal of Cosmetic Dermatology. 2022;21(10):5203-7.
196. Leinweber B, Massone C, Kodama K, Kaddu S, Cerroni L, Haas J, et al. Teledermatopathology: A controlled study about diagnostic validity and technical requirements for digital transmission. American Journal of Dermatopathology. 2006;28(5):413-6.
197. Li FF, Wang M, Wang T, Wang XL, Ma XL, He H, et al. Smartphone-based infrared thermography to assess progress in thoracic surgical incision healing: A preliminary study. International Wound Journal. 2023;20(6):2000-9.
198. Li TH, Wang JD, Xie HY, Hao PS, Qing C, Zhang YZ, et al. Study on the related factors of post-herpetic neuralgia in hospitalized patients with herpes zoster in Sichuan Hospital of Traditional Chinese Medicine based on big data analysis. Dermatologic Therapy. 2020;33(6):e14410.
199. Lim K, Neal-Smith G, Mitchell C, Xerri J, Chuanromanee P. Perceptions of the use of artificial intelligence in the diagnosis of skin cancer: an outpatient survey. Clinical and Experimental Dermatology. 2022;47(3):542-6.
200. Lim ZV, Akram F, Ngo CP, Winarto AA, Lee WQ, Liang KC, et al. Automated grading of acne vulgaris by deep learning with convolutional neural networks. Skin Research and Technology. 2020;26(2):187-92.
201. Liopyris K, Gregoriou S, Dias J, Stratigos AJ. Artificial Intelligence in Dermatology: Challenges and Perspectives. Dermatology and Therapy. 2022;12(12):2637-51.
202. Liu L, Liang C, Xue YZ, Chen TQ, Chen YM, Lan YF, et al. An Intelligent Diagnostic Model for Melasma Based on Deep Learning and Multimode Image Input. Dermatology and Therapy. 2023;13(2):569-79.
203. Liu NT, Rizzo JA, Shields BA, Serio-Melvin ML, Christy RJ, Salinas J. Predicting the Ability of Wounds to Heal Given Any Burn Size and Fluid Volume: An Analytical Approach. Journal of Burn Care & Research. 2018;39(5):661-9.
204. Liu NT, Salinas J. Machine learning in burn care and research: A systematic review of the literature. Burns. 2015;41(8):1636-41.
205. Liu S, Fan YS, Duan MY, Wang YY, Su GX, Ren YJ, et al. AcneGrader: An ensemble pruning of the deep learning base models to grade acne. Skin Research and Technology. 2022;28(5):677-88.
206. Lowenstein EJ, Sidlow R. Diagnostic heuristics in dermatology, part 2: metacognition and other fixes. British Journal of Dermatology. 2018;179(6):1270-6.
207. Luboz V, Bailet M, Grivot CB, Rochette M, Diot B, Bucki M, et al. Personalized modeling for real-time pressure ulcer prevention in sitting posture. Journal of Tissue Viability. 2018;27(1):54-8.
208. Lucas Y, Niri R, Treuillet S, Douzi H, Castaneda B. Wound Size Imaging: Ready for Smart Assessment and Monitoring. Advances in Wound Care. 2021;10(11):641-61.
209. Lunge SB, Shetty NS, Sardesai VR, Karagaiah P, Yamauchi PS, Weinberg JM, et al. Therapeutic application of machine learning in psoriasis: A Prisma systematic review. Journal of Cosmetic Dermatology. 2023;22(2):378-82.
210. Lunter D, Klang V, Kocsis D, Varga-Medveczky Z, Berko S, Erdo F. Novel aspects of Raman spectroscopy in skin research. Experimental Dermatology. 2022;31(9):1311-29.
211. Lustig A, Gefen A. Three-dimensional shape-conformation performances of wound dressings tested in a robotic sacral pressure ulcer phantom. International Wound Journal. 2021;18(5):670-80.
212. Lustig A, Gefen A. Fluid management and strength postsimulated use of primary and secondary dressings for treating diabetic foot ulcers: Robotic phantom studies. International Wound Journal. 2022;19(2):305-15.
213. Lustig A, Gefen A. The performance of gelling fibre wound dressings under clinically relevant robotic laboratory tests. International Wound Journal. 2022;19:3-21.
214. Lustig M, Schwartz D, Bryant R, Gefen A. A machine learning algorithm for early detection of heel deep tissue injuries based on a daily history of sub-epidermal moisture measurements. International Wound Journal. 2022;19(6):1339-48.
215. MacLellan AN, Price EL, Publicover-Brouwer P, Matheson K, Ly TY, Pasternak S, et al. The use of noninvasive imaging techniques in the diagnosis of melanoma: a prospective diagnostic accuracy study. Journal of the American Academy of Dermatology. 2021;85(2):353-9.
216. Magalhaes C, Mendes J, Vardasca R. The role of AI classifiers in skin cancer images. Skin Research and Technology. 2019;25(5):750-7.
217. Maintz L, Welchowski T, Herrmann N, Brauer J, Klaschen AS, Fimmers R, et al. Machine Learning-Based Deep Phenotyping of Atopic Dermatitis Severity-Associated Factors in Adolescent and Adult Patients. Jama Dermatology. 2021;157(12):1414-24.
218. Majidian M, Tejani I, Jarmain T, Kellett L, Moy R. Artificial Intelligence in the Evaluation of Telemedicine Dermatology Patients. Journal of Drugs in Dermatology. 2022;21(2):83-6.
219. Majumder SK, Ghosh N, Gupta PK. Relevance vector machine for optical diagnosis of cancer. Lasers in Surgery and Medicine. 2005;36(4):323-33.
220. Marchetti MA, Codella NCF, Dusza SW, Gutman DA, Helba B, Kalloo A, et al. Results of the 2016 International Skin Imaging Collaboration International Symposium on Biomedical Imaging challenge: Comparison of the accuracy of computer algorithms to dermatologists for the diagnosis of melanoma from dermoscopic images. Journal of the American Academy of Dermatology. 2018;78(2):270-7.
221. Marchetti MA, Liopyris K, Dusza SW, Codella NCF, Gutman DA, Helba B, et al. Computer algorithms show potential for improving dermatologists' accuracy to diagnose cutaneous melanoma: Results of the International Skin Imaging Collaboration 2017. Journal of the American Academy of Dermatology. 2020;82(3):622-7.
222. Marghoob AA, Swindle LD, Moricz CZM, Negron FAS, Slue B, Halpern AC, et al. Instruments and new technologies for the in vivo diagnosis of melanoma. Journal of the American Academy of Dermatology. 2003;49(5):777-97.
223. Margolis DJ, Mitra N, Malay DS, Mirza ZK, Lantis JC, Lev-Tov HA, et al. Further evidence that wound size and duration are strong prognostic markers of diabetic foot ulcer healing. Wound Repair and Regeneration. 2022;30(4):487-90.
224. Martorell A, Martin-Gorgojo A, Rios-Vinuela E, Rueda-Carnero JM, Alfageme F, Taberner R. Artificial Intelligence in Dermatology: A Threat or an Opportunity? Actas Dermo-Sifiliograficas. 2022;113(1):30-46.
225. Massone C, Brunasso AMG, Campbell TM, Soyer HP. State of the art of teledermatopathology. American Journal of Dermatopathology. 2008;30(5):446-50.
226. Mathur J, Chouhan V, Pangti R, Kumar S, Gupta S. A convolutional neural network architecture for the recognition of cutaneous manifestations of COVID-19. Dermatologic Therapy. 2021;34(2): e14902.
227. Maurya A, Stanley RJ, Lama N, Jagannathan S, Saeed D, Swinfard S, et al. A deep learning approach to detect blood vessels in basal cell carcinoma. Skin Research and Technology. 2022;28(4):571-6.
228. Mehrabi JN, Baugh EG, Fast A, Lentsch G, Balu M, Lee BA, et al. A Clinical Perspective on the Automated Analysis of Reflectance Confocal Microscopy in Dermatology. Lasers in Surgery and Medicine. 2021;53(8):1011-9.
229. Meienberger N, Anzengruber F, Amruthalingam L, Christen R, Koller T, Maul JT, et al. Observer-independent assessment of psoriasis-affected area using machine learning. Journal of the European Academy of Dermatology and Venereology. 2020;34(6):1362-8.
230. Melina A, Dinh NN, Tafuri B, Schipani G, Nistico S, Cosentino C, et al. Artificial Intelligence for the Objective Evaluation of Acne Investigator Global Assessment. Journal of Drugs in Dermatology. 2018;17(9):1006-9.
231. Messaraa C, Richard TJC, Walsh M, Doyle L, O'Connor C, Robertson N, et al. Perceived age and perceived health among a Chinese cohort: Does it mean the same thing? International Journal of Cosmetic Science. 2020;42(5):471-81.
232. Mikolajczyk M, Patrzyk S, Nieniewski M, Wozniacka A. Evaluation of a smartphone application for diagnosis of skin diseases. Postepy Dermatologii I Alergologii. 2021;38(5):761-6.
233. Minagawa A, Koga H, Sano T, Matsunaga K, Teshima Y, Hamada A, et al. Dermoscopic diagnostic performance of Japanese dermatologists for skin tumors differs by patient origin: A deep learning convolutional neural network closes the gap. Journal of Dermatology. 2021;48(2):232-6.
234. Moglia A, Cerri A, Berchiolli R, Ferrari M, Betti R. Machine learning for the identification of decision boundaries during the transition from radial to vertical growth phase superficial spreading melanomas. Melanoma Research. 2021;31(6):533-40.
235. Mohr P, Birgersson U, Berking C, Henderson C, Trefzer U, Kemeny L, et al. Electrical impedance spectroscopy as a potential adjunct diagnostic tool for cutaneous melanoma. Skin Research and Technology. 2013;19(2):75-83.
236. Monheit G, Cognetta AB, Ferris L, Rabinovitz H, Gross K, Martini M, et al. The Performance of MelaFind A Prospective Multicenter Study. Archives of Dermatology. 2011;147(2):188-94.
237. Moura FSE, Amin K, Ekwobi C. Artificial intelligence in the management and treatment of burns: a systematic review. Burns & Trauma. 2021;9:tkab022.
238. Munger E, Choi H, Dey AK, Elnabawi YA, Groenendyk JW, Rodante J, et al. Application of machine learning to determine top predictors of noncalcified coronary burden in psoriasis: An observational cohort study. Journal of the American Academy of Dermatology. 2020;83(6):1647-53.
239. Munoz-Lopez C, Ramirez-Cornejo C, Marchetti MA, Han SS, Del Barrio-Diaz P, Jaque A, et al. Performance of a deep neural network in teledermatology: a single-centre prospective diagnostic study. Journal of the European Academy of Dermatology and Venereology. 2021;35(2):546-53.
240. Nagaoka T, Nakamura A, Okutani H, Kiyohara Y, Sota T. A possible melanoma discrimination index based on hyperspectral data: a pilot study. Skin Research and Technology. 2012;18(3):301-10.
241. Nagata T, Noyori SS, Noguchi H, Nakagami G, Kitamura A, Sanada H. Skin tear classification using machine learning from digital RGB image. Journal of Tissue Viability. 2021;30(4):588-93.
242. Nakamura M, Haarmann-Stemmann T, Krutmann J, Morita A. Alternative test models for skin ageing research. Experimental Dermatology. 2018;27(5):495-500.
243. Narbutt J, Krzyscin J, Sobolewski P, Skibinska M, Noweta M, Owczarek W, et al. A Priori Estimation of the Narrow-Band UVB Phototherapy Outcome for Moderate-to-Severe Psoriasis Based on the Patients' Questionnaire and Blood Tests Using Random Forest Classifier. Clinical Cosmetic and Investigational Dermatology. 2021;14:253-9.
244. Narla S, Heath CR, Alexis A, Silverberg JI. Racial disparities in dermatology. Archives of Dermatological Research. 2023;315(5):1215-23.
245. Nelson CA, Perez-Chada LM, Creadore A, Li SJ, Lo K, Manjaly P, et al. Patient Perspectives on the Use of Artificial Intelligence for Skin Cancer Screening A Qualitative Study. Jama Dermatology. 2020;156(5):501-12.
246. Nielsen ML, Petersen TC, Maul JT, Wu JJ, Rasmussen MK, Bertelsen T, et al. Multivariable Predictive Models to Identify the Optimal Biologic Therapy for Treatment of Patients With Psoriasis at the Individual Level. Jama Dermatology. 2022;158(10):1149-56.
247. Ocagli H, Lorenzoni G, Bottigliengo D, Azzolina D, Stivanello L, Giorato E, et al. The SYSTEMIC Project: A Pilot Study to Develop a Registry of Patients With an Ostomy for Predictive Modeling of Outcomes. Wound Management & Prevention. 2021;67(4):24-34.
248. Ogrin R, Motin MA, Aliahmad B, Elder K, Anderson J, Kumar D. Can Thermal Imaging Technique be Used to Predict the Healing Status of a Venous Leg Ulcer? International Journal of Lower Extremity Wounds. 2023;22(1):85-92.
249. Ohura N, Mitsuno R, Sakisaka M, Terabe Y, Morishige Y, Uchiyama A, et al. Convolutional neural networks for wound detection: the role of artificial intelligence in wound care. Journal of Wound Care. 2019;28(10):S13-S24.
250. Okamoto T, Kawai M, Ogawa Y, Shimada S, Kawamura T. Artificial intelligence for the automated single-shot assessment of psoriasis severity. Journal of the European Academy of Dermatology and Venereology. 2022;36(12):2512-5.
251. Okon E, Rachakonda V, Hong HJ, Callison-Burch C, Lipoff JB. Natural language processing of Reddit data to evaluate dermatology patient experiences and therapeutics. Journal of the American Academy of Dermatology. 2020;83(3):803-8.
252. Onuh OC, Brydges HT, Nasr H, Savage E, Gorenstein S, Chiu E. Capturing Essentials in Wound Photography Past, Present, and Future: A Proposed Algorithm for Standardization. Advances in Skin & Wound Care. 2022;35(9):483-92.
253. Orlov A, Gefen A. The fluid handling performance of the curea P1 multipurpose dressing against superabsorbent and foam dressing technologies. International Wound Journal. 2022;19(4):945-56.
254. Orlov A, Gefen A. Fluid handling performance of wound dressings tested in a robotic venous leg ulcer system under compression therapy. International Wound Journal. 2023;20(5):1384-92.
255. Orlov A, Lustig A, Grigatti A, Gefen A. Fluid Handling Dynamics and Durability of Silver-Containing Gelling Fiber Dressings Tested in a Robotic Wound System. Advances in Skin & Wound Care. 2022;35(6):326-34.
256. Oukil S, Kasmi R, Mokrani K, Garcia-Zapirain B. Automatic segmentation and melanoma detection based on color and texture features in dermoscopic images. Skin Research and Technology. 2022;28(2):203-11.
257. Pai VV, Pai RB. Artificial intelligence in dermatology and healthcare: An overview. Indian Journal of Dermatology Venereology & Leprology. 2021;87(4):457-67.
258. Pak HS. Teledermatology and teledermatopathology. Seminars in Cutaneous Medicine and Surgery. 2002;21(3):179-89.
259. Pangti R, Mathur J, Chouhan V, Kumar S, Rajput L, Shah S, et al. A machine learning-based, decision support, mobile phone application for diagnosis of common dermatological diseases. Journal of the European Academy of Dermatology and Venereology. 2021;35(2):536-45.
260. Papp KA, Soliman AM, Done N, Carley C, Wirtz EL, Puig L. Deterioration of Health-Related Quality of Life After Withdrawal of Risankizumab Treatment in Patients with Moderate-to-Severe Plaque Psoriasis: A Machine Learning Predictive Model. Dermatology and Therapy. 2021;11(4):1291-304.
261. Parad JE, Liao W. Bioinformatic applications in psoriasis: genetics, transcriptomics, and microbiomics. Seminars in Cutaneous Medicine and Surgery. 2019;38(1):E3-E11.
262. Park E, Kwon KH. A popularization of curation service for dermatological condition in Republic of Korea. Journal of Cosmetic Dermatology. 2022;21(12):6594-604.
263. Pasquali P, Sonthalia S, Moreno-Ramirez D, Sharma P, Agrawal M, Gupta S, et al. Teledermatology and its current perspective. Indian Dermatology Online Journal. 2020;11(1):12-20.
264. Patel JK, Konda S, Perez OA, Amini S, Elgart G, Berman B. Newer technologies/techniques and tools in the diagnosis of melanoma. European Journal of Dermatology. 2008;18(6):617-31.
265. Patel S, Wang JV, Motaparthi K, Lee JB. Artificial intelligence in dermatology for the clinician. Clinics in Dermatology. 2021;39(4):667-72.
266. Patil S, Rao ND, Patil A, Basar F, Bate S. Assessment of tibot (R) artificial intelligence application in prediction of diagnosis in dermatological conditions: results of a single centre study. Indian Dermatology Online Journal. 2020;11(6):910-4.
267. Patrick MT, Raja K, Miller K, Sotzen J, Gudjonsson JE, Elder JT, et al. Drug Repurposing Prediction for Immune-Mediated Cutaneous Diseases using a Word-Embedding-Based Machine Learning Approach. Journal of Investigative Dermatology. 2019;139(3):683-91.
268. Patrzyk S, Bielecki W, Wozniacka A. A study of attitudes among Polish dermatologists and dermatology trainees regarding modern technologies in medicine. Postepy Dermatologii I Alergologii. 2022;39(3):531-7.
269. Patterson DR, Drever S, Soltani M, Sharar SR, Wiechman S, Meyer WJ, et al. A comparison of interactive immersive virtual reality and still nature pictures as distraction- based analgesia in burn wound care. Burns. 2023;49(1):182-92.
270. Pellacani G, Grana C, Cucchiara R, Seidenari S. Automated extraction and description of dark areas in surface microscopy melanocytic lesion images. Dermatology. 2004;208(1):21-6.
271. Pereira JC, Pereira JPC. Megasessions for Robotic Hair Restoration. Journal of Drugs in Dermatology. 2016;15(11):1407-12.
272. Petrie T, Samatham R, Witkowski AM, Esteva A, Leachman SA. Melanoma Early Detection: Big Data, Bigger Picture. Journal of Investigative Dermatology. 2019;139(1):25-30.
273. Phelan HA, Iv JHH, Hickerson WL, Cockerell CJ, Shupp JW, Carter JE. Use of 816 Consecutive Burn Wound Biopsies to Inform a Histologic Algorithm for Burn Depth Categorization. Journal of Burn Care & Research. 2021;42(6):1162-7.
274. Phillips M, Greenhalgh J, Marsden H, Palamaras I. Detection of Malignant Melanoma Using Artificial Intelligence: An Observational Study of Diagnostic Accuracy. Dermatology Practical & Conceptual. 2020;10(1):e1913436.
275. Piccolo D, Ferrari A, Peris K, Daidone R, Ruggeri B, Chimenti S. Dermoscopic diagnosis by a trained clinician vs. a clinician with minimal dermoscopy training vs. computer-aided diagnosis of 341 pigmented skin lesions: a comparative study. British Journal of Dermatology. 2002;147(3):481-6.
276. Pinter A, Puig L, Schakel K, Reich A, Zaheri S, Costanzo A, et al. Comparative effectiveness of biologics in clinical practice: week 12 primary outcomes from an international observational psoriasis study of health outcomes (PSoHO). Journal of the European Academy of Dermatology and Venereology. 2022;36(11):2087-100.
277. Pivneva I, Balp MM, Geissbuhler Y, Severin T, Smeets S, Signorovitch J, et al. Predicting Clinical Remission of Chronic Urticaria Using Random Survival Forests: Machine Learning Applied to Real-World Data. Dermatology and Therapy. 2022;12(12):2747-63.
278. Polesie S, Gillstedt M, Kittler H, Rinner C, Tschandl P, Paoli J. Assessment of melanoma thickness based on dermoscopy images: an open, web-based, international, diagnostic study. Journal of the European Academy of Dermatology and Venereology. 2022;36(11):2002-7.
279. Psaty EL, Halpern AC. Current and emerging technologies in melanoma diagnosis: the state of the art. Clinics in Dermatology. 2009;27(1):35-45.
280. Quattrini A, Boer C, Leidi T, Paydar R. A Deep Learning-Based Facial Acne Classification System. Clinical Cosmetic and Investigational Dermatology. 2022;15:851-7.
281. Rajpara SM, Botello AP, Townend J, Ormerod AD. Systematic review of dermoscopy and digital dermoscopy/artificial intelligence for the diagnosis of melanoma. British Journal of Dermatology. 2009;161(3):591-604.
282. Rangaraju LP, Kunapuli G, Every D, Ayala OD, Ganapathy P, Mahadevan-Jansen A. Classification of burn injury using Raman spectroscopy and optical coherence tomography: An ex-vivo study on porcine skin. Burns. 2019;45(3):659-70.
283. Rasanen J, Salmivuori M, Polonen I, Gronroos M, Neittaanmaki N. Hyperspectral Imaging Reveals Spectral Differences and Can Distinguish Malignant Melanoma from Pigmented Basal Cell Carcinomas: A Pilot Study. Acta Dermato-Venereologica. 2021;101:adv00405.
284. Reiter O, Rotemberg V, Kose K, Halpern AC. Artificial Intelligence in Skin Cancer. Current Dermatology Reports. 2019;8(3):133-40.
285. Ring C, Cox N, Lee JB. Dermatoscopy. Clinics in Dermatology. 2021;39(4):635-42.
286. Robb L. Potential for Machine Learning in Burn Care. Journal of Burn Care & Research. 2022;43(3):632-9.
287. Rose PT. Advances in Hair Restoration. Dermatologic Clinics. 2018;36(1):57-62.
288. Rose PT, Nusbaum B. Robotic Hair Restoration. Dermatologic Clinics. 2014;32(1):97-107.
289. Ross M, Watcher MA, Goodman MM. Comparison of the flashlamp pulsed dye laser with the argon tunable dye-laser with robotized handpiece for facial telangiectasia. Lasers in Surgery and Medicine. 1993;13(3):374-8.
290. Rotemberg V, Halpern A, Dusza S, Codella NCF. The role of public challenges and data sets towards algorithm development, trust, and use in clinical practice. Seminars in Cutaneous Medicine and Surgery. 2019;38(1):E38-E42.
291. Rotteleur G, Mordon S, Sozanski JP, Brunetaud JM. Robotized scanning laser hand piece for the treatment of port wine stains and other angiodysplasias. Lasers in Surgery and Medicine. 1988;8(3):283-7.
292. Rubegni P, Burroni M, Cevenini G, Perotti R, Dell'Eva G, Barbini P, et al. Digital dermoscopy analysis and artificial neural network for the differentiation of clinically atypical pigmented skin lesions: A retrospective study. Journal of Investigative Dermatology. 2002;119(2):471-4.
293. Rubegni P, Cevenini G, Flori ML, Barbini P, Andreassi L. Relationship between minimal phototoxic dose and skin colour plus sun exposure history: a neural network approach. Photodermatology Photoimmunology & Photomedicine. 1998;14(1):26-30.
294. Rubegni P, Cevenini G, Nami N, Argenziano G, Saida T, Burroni M, et al. Dermoscopy and Digital Dermoscopy Analysis of Palmoplantar 'Equivocal' Pigmented Skin Lesions in Caucasians. Dermatology. 2012;225(3):248-55.
295. Rubegni P, Feci L, Nami N, Burroni M, Taddeucci P, Miracco C, et al. Computer-assisted melanoma diagnosis: a new integrated system. Melanoma Research. 2015;25(6):537-42.
296. Rundle CW, Hollingsworth P, Dellavalle RP. Artificial intelligence in dermatology. Clinics in Dermatology. 2021;39(4):657-66.
297. Sacha JP, Caterino TL, Fisher BK, Carr GJ, Youngquist RS, D'Alessandro BM, et al. Development and qualification of a machine learning algorithm for automated hair counting. International Journal of Cosmetic Science. 2021;43:S34-S41.
298. Sachar M, Xiong M, Lee KC. Consumer preferences of top-rated over-the-counter acne treatment products: a cohort study. Archives of Dermatological Research. 2022;314(8):815-21.
299. Sadeghi M, Chilana P, Yap J, Tschandl P, Atkins MS. Using content-based image retrieval of dermoscopic images for interpretation and education: A pilot study. Skin Research and Technology. 2020;26(4):503-12.
300. Sadick NS. New-Generation Therapies for the Treatment of Hair Loss in Men. Dermatologic Clinics. 2018;36(1):63-7.
301. Samaran R, L'Orphelin JM, Dreno B, Rat C, Dompmartin A. Interest in artificial intelligence for the diagnosis of non-melanoma skin cancer: a survey among French general practitioners. European Journal of Dermatology. 2021;31(4):457-62.
302. Samhan AF, Abdelhalim NM, Elnaggar RK. Effects of interactive robot-enhanced hand rehabilitation in treatment of paediatric hand-burns: A randomized, controlled trial with 3-months follow-up. Burns. 2020;46(6):1347-55.
303. Sangers TE, Wakkee M, Kramer-Noels EC, Nijsten T, Lugtenberg M. Views on mobile health apps for skin cancer screening in the general population: an in-depth qualitative exploration of perceived barriers and facilitators. British Journal of Dermatology. 2021;185(5):961-9.
304. Sangers TE, Wakkee M, Moolenburgh FJ, Nijsten T, Lugtenberg M. Towards successful implementation of artificial intelligence in skin cancer care: a qualitative study exploring the views of dermatologists and general practitioners. Archives of Dermatological Research. 2023;315(5):1187-95.
305. Sangers T, Reeder S, van der Vet S, Jhingoer S, Mooyaart A, Siegel DM, et al. Validation of a Market-Approved Artificial Intelligence Mobile Health App for Skin Cancer Screening: A Prospective Multicenter Diagnostic Accuracy Study. Dermatology. 2022;238(4):649-56.
306. Savolainen L, Kontinen J, Alatalo E, Roning J, Oikarinen A. Comparison of actual psoriasis surface area and the psoriasis area and severity index by the human eye and machine vision methods in following the treatment of psoriasis. Acta Dermato-Venereologica. 1998;78(6):466-7.
307. Schaap MJ, Cardozo NJ, Patel A, De Jong E, Van Ginneken B, Seyger MMB. Image-based automated Psoriasis Area Severity Index scoring by Convolutional Neural Networks. Journal of the European Academy of Dermatology and Venereology. 2022;36(1):68-75.
308. Schafrank LA, Falkner RC, Lam TK, Meyerle JH. Teledermatology in Military Settings. Current Dermatology Reports. 2021;10(2):33-9.
309. Schindewolf T, Schiffner R, Stolz W, Albert R, Abmayr W, Harms H. Evaluation of different image acquisition techniques for a computer vision system in the diagnosis of malignant melanoma. Journal of the American Academy of Dermatology. 1994;31(1):33-41.
310. 310. Schlessinger DI, Chhor G, Gevaert O, Swetter SM, Ko J, Novoa RA. Artificial intelligence and dermatology: opportunities, challenges, and future directions. Seminars in Cutaneous Medicine and Surgery. 2019;38(1):E31-E7.
311. 311. Schmelter V, Hofmann T, Schneider F, Weber C, Fuerweger C, Muacevic A, et al. Robotic CyberKnife radiosurgery for small choroidal melanomas. Melanoma Research. 2022;32(3):192-9.
312. 312. Schmid-Grendelmeier P, Takaoka R, Ahogo KC, Belachew WA, Brown SJ, Correia JC, et al. Position Statement on Atopic Dermatitis in Sub-Saharan Africa: current status and roadmap. Journal of the European Academy of Dermatology and Venereology. 2019;33(11):2019-28.
313. Schneider SL, Kohli I, Hamzavi IH, Council ML, Rossi AM, Ozog DM. Emerging imaging technologies in dermatology Part I: Basic principles. Journal of the American Academy of Dermatology. 2019;80(4):1114-20.
314. Schneider SL, Kohli I, Hamzavi IH, Council ML, Rossi AM, Ozog DM. Emerging imaging technologies in dermatology Part II: Applications and limitations. Journal of the American Academy of Dermatology. 2019;80(4):1121-31.
315. Seite S, Khammari A, Benzaquen M, Moyal D, Dreno B. Development and accuracy of an artificial intelligence algorithm for acne grading from smartphone photographs. Experimental Dermatology. 2019;28(11):1252-7.
316. Sendin-Martin M, Lara-Caro M, Harris U, Moronta M, Rossi A, Lee E, et al. Classification of Basal Cell Carcinoma in Ex Vivo Confocal Microscopy Images from Freshly Excised Tissues Using a Deep Learning Algorithm. Journal of Investigative Dermatology. 2022;142(5):1291-9.
317. Sengupta S, Mittal N, Modi M. Improved skin lesions detection using color space and artificial intelligence techniques. Journal of Dermatological Treatment. 2020;31(5):511-8.
318. Seo JI, Ham HI, Baek JH, Shin MK. An objective skin-type classification based on non-invasive biophysical parameters. Journal of the European Academy of Dermatology and Venereology. 2022;36(3):444-52.
319. Shah BM, Ganvir D, Sharma YK, Mirza SB, Misra RN, Kothari P, et al. Utility of a real-time fluorescence imaging device in guiding antibiotic treatment in superficial skin infections. Indian Journal of Dermatology Venereology & Leprology. 2022;88(4):509-14.
320. Sharma AN, Shwe S, Mesinkovska NA. Current state of machine learning for non-melanoma skin cancer. Archives of Dermatological Research. 2022;314(4):325-7.
321. Sharma A, Jindal V, Singla P, Goldust M, Mhatre M. Will teledermatology be the silver lining during and after COVID-19? Dermatologic Therapy. 2020;33(4):e13643.
322. Shim J, Lim JM, Park SG. Machine learning for the prediction of sunscreen sun protection factor and protection grade of UVA. Experimental Dermatology. 2019;28(7):872-4.
323. Shin JW, Kwon SH, Kim SA, Kim JY, Na JI, Park KC, et al. Characteristics of robotically harvested hair follicles in Koreans. Journal of the American Academy of Dermatology. 2015;72(1):146-50.
324. Shoham G, Berl A, Shir-Az O, Shabo S, Shalom A. Predicting Mohs surgery complexity by applying machine learning to patient demographics and tumor characteristics. Experimental Dermatology. 2022;31(7):1029-35.
325. Silver FH, Deshmukh T, Kelkar N, Ritter K, Ryan N, Nadiminti H. The "Virtual Biopsy" of Cancerous Lesions in 3D: Non-Invasive Differentiation between Melanoma and Other Lesions Using Vibrational Optical Coherence Tomography. Dermatopathology. 2021;8(4):539-51.
326. Singh Y. The Application of Machine Learning in Predicting Outcome of Cryotherapy and Immunotherapy for Wart Removal. Annals of Dermatology. 2021;33(4):345-50.
327. Skudalski L, Waldman R, Kerr PE, Grant-Kels JM. Melanoma: How and when to consider clinical diagnostic technologies. Journal of the American Academy of Dermatology. 2022;86(3):503-12.
328. Smith MP, Ly K, Thibodeaux Q, Weerasinghe T, Wu JJ, Yosipovitch G, et al. Emerging Methods to Objectively Assess Pruritus in Atopic Dermatitis. Dermatology and Therapy. 2019;9(3):407-20.
329. Snyder AN, Zhang D, Dreesen SL, Baltimore CA, Lopez-Garcia DR, Akers JY, et al. Histologic Screening of Malignant Melanoma, Spitz, Dermal and Junctional Melanocytic Nevi Using a Deep Learning Model. American Journal of Dermatopathology. 2022;44(9):650-7.
330. Sokolov K, Shpudeiko V. Dynamics of the Neural Network Accuracy in the Context of Modernization of the Algorithms of Skin Pathology Recognition. Indian Journal of Dermatology. 2022;67(3):312.
331. Song JY, Woo K, Shang JJ, Ojo M, Topaz M. Predictive Risk Models for Wound Infection-Related Hospitalization or ED Visits in Home Health Care Using Machine-Learning Algorithms. Advances in Skin & Wound Care. 2021;34(8):1-12.
332. Speiser JJ, Hughes I, Mehta V, Wojcik EM, Hutchens KA. Mobile Teledermatopathology: Using a Tablet PC as a Novel and Cost-Efficient Method to Remotely Diagnose Dermatopathology Cases. American Journal of Dermatopathology. 2014;36(1):54-7.
333. Stiff KM, Franklin MJ, Zhou YF, Madabhushi A, Knackstedt TJ. Artificial intelligence and melanoma: A comprehensive review of clinical, dermoscopic, and histologic applications. Pigment Cell & Melanoma Research. 2022;35(2):203-11.
334. Stojkovic-Filipovic J, Tiodorovic D, Lallas A, Akay BN, Longo C, Rosendahl C, et al. Dermatoscopy of combined blue nevi: a multicentre study of the International Dermoscopy Society. Journal of the European Academy of Dermatology and Venereology. 2021;35(4):900-5.
335. Stylianou N, Akbarov A, Kontopantelis E, Buchan I, Dunn KW. Mortality risk prediction in burn injury: Comparison of logistic regression with machine learning approaches. Burns. 2015;41(5):925-34.
336. Sultana N. Predicting sun protection measures against skin diseases using machine learning approaches. Journal of Cosmetic Dermatology. 2022;21(2):758-69.
337. Sun JC, Fu L, Zhang W, Li DJ, Zhang M, Xu ZN, et al. Convolutional neural network models for automatic diagnosis and graduation in skin frostbite. International Wound Journal. 2023;20(4):910-6.
338. Sun MD, Halpern AC. Advances in the Etiology, Detection, and Clinical Management of Seborrheic Keratoses. Dermatology. 2022;238(2):205-17.
339. Sun MD, Kentley J, Wilson BW, Soyer HP, Curiel-Lewandrowski CN, Rotemberg VM, et al. Digital skin imaging applications, part II: a comprehensive survey of post-acquisition image utilization features and technology standards. Skin Research and Technology. 2022;28(6):771-9.
340. Sun MD, Kentley J, Wilson BW, Soyer HP, Curiel-Lewandrowski CN, Rotemberg V, et al. Digital skin imaging applications, part I: Assessment of image acquisition technique features. Skin Research and Technology. 2022;28(4):623-32.
341. Sutaria N, Alphonse MP, Marani M, Parthasarathy V, Deng JW, Wongvibulsin S, et al. Cluster Analysis of Circulating Plasma Biomarkers in Prurigo Nodularis Reveals a Distinct Systemic Inflammatory Signature in African Americans. Journal of Investigative Dermatology. 2022;142(5):1300-8.
342. Talebi-Liasi F, Markowitz O. Is Artificial Intelligence Going to Replace Dermatologists? Cutis. 2020;105(1):28-31.
343. Tan E, Lin F, Sheck L, Salmon P, Ng S. A practical decision-tree model to predict complexity of reconstructive surgery after periocular basal cell carcinoma excision. Journal of the European Academy of Dermatology and Venereology. 2017;31(4):717-23.
344. Taroni JN, Martyanov V, Mahoney JM, Whitfield ML. A Functional Genomic Meta-Analysis of Clinical Trials in Systemic Sclerosis: Toward Precision Medicine and Combination Therapy. Journal of Investigative Dermatology. 2017;137(5):1033-41.
345. Taylor M, Liu XX, Denniston A, Esteva A, Ko J, Daneshjou R, et al. Raising the Bar for Randomized Trials Involving Artificial Intelligence: The SPIRIT-Artificial Intelligence and CONSORT-Artificial Intelligence Guidelines. Journal of Investigative Dermatology. 2021;141(9):2109-11.
346. Tenenhaus A, Nkengne A, Horn JF, Serruys C, Giron A, Fertil B. Detection of melanoma from dermoscopic images of naevi acquired under uncontrolled conditions. Skin Research and Technology. 2010;16(1):85-97.
347. Thomsen K, Iversen L, Titlestad TL, Winther O. Systematic review of machine learning for diagnosis and prognosis in dermatology. Journal of Dermatological Treatment. 2020;31(5):496-510.
348. Tkaczyk ER, Coco JR, Wang JN, Chen FY, Ye C, Jagasia MH, et al. Crowdsourcing to delineate skin affected by chronic graft-vs-host disease. Skin Research and Technology. 2019;25(4):572-7.
349. Tognetti L, Bonechi S, Andreini P, Bianchini M, Scarselli F, Cevenini G, et al. A new deep learning approach integrated with clinical data for the dermoscopic differentiation of early melanomas from atypical nevi. Journal of Dermatological Science. 2021;101(2):115-22.
350. Tomalin LE, Kim J, da Rosa JC, Lee J, Fitz LJ, Berstein G, et al. Early Quantification of Systemic Inflammatory Proteins Predicts Long-Term Treatment Response to Tofacitinib and Etanercept. Journal of Investigative Dermatology. 2020;140(5):1026-34.
351. Toncic RJ, Jakasa I, Sun Y, Hurault G, Hadzavdic SL, Tanaka RJ, et al. Stratum corneum markers of innate and T helper cell-related immunity and their relation to the disease severity in Croatian patients with atopic dermatitis. Journal of the European Academy of Dermatology and Venereology. 2021;35(5):1186-96.
352. Torres R, Lang UE, Hejna M, Shelton SJ, Joseph NM, Shain AH, et al. MicroRNA Ratios Distinguish Melanomas from Nevi. Journal of Investigative Dermatology. 2020;140(1):164-73.
353. Tran NK, Sen S, Palmieri TL, Lima K, Falwell S, Wajda J, et al. Artificial intelligence and machine learning for predicting acute kidney injury in severely burned patients: A proof of concept. Burns. 2019;45(6):1350-8.
354. Tschandl P. Artificial intelligence for melanoma diagnosis. Italian Journal of Dermatology and Venereology. 2021;156(3):289-99.
355. Udrea A, Mitra GD, Costea D, Noels EC, Wakkee M, Siegel DM, et al. Accuracy of a smartphone application for triage of skin lesions based on machine learning algorithms. Journal of the European Academy of Dermatology and Venereology. 2020;34(3):648-55.
356. Uppal SK, Beer J, Hadeler E, Gitlow H, Nouri K. The clinical utility of teledermoscopy in the era of telemedicine. Dermatologic Therapy. 2021;34(2).
357. Urban G, Feil N, Csuka E, Hashemi K, Ekelem C, Choi F, et al. Combining Deep Learning With Optical Coherence Tomography Imaging to Determine Scalp Hair and Follicle Counts. Lasers in Surgery and Medicine. 2021;53(1):171-8.
358. Van Neste D, Trueb RM. Critical study of hair growth analysis with computer-assisted methods. Journal of the European Academy of Dermatology and Venereology. 2006;20(5):578-83.
359. van Zon MCM, van der Waa JD, Veta M, Krekels GAM. Whole-slide margin control through deep learning in Mohs micrographic surgery for basal cell carcinoma. Experimental Dermatology. 2021;30(5):733-8.
360. Velickovic VM, Spelman T, Clark M, Probst S, Armstrong DG, Steyerberg E. Individualized Risk Prediction for Improved Chronic Wound Management. Advances in Wound Care. 2023;12(7):387-98.
361. Verma N, Singh S, Roy A, Valsan A, Garg P, Pradhan P, et al. Cirrhosis and fungal infections-a cocktail for catastrophe: A systematic review and meta-analysis with machine learning. Mycoses. 2022;65(9):844-58.
362. Viljanto J, Koski A. Neural network adapted to wound cell analysis in surgical patients. Wound Repair and Regeneration. 2011;19(2):162-7.
363. Viswanathan V, Govindan S, Selvaraj B, Rupert S, Kumar R. A Clinical Study to Evaluate Autofluorescence Imaging of Diabetic Foot Ulcers Using a Novel Artificial Intelligence Enabled Noninvasive Device. International Journal of Lower Extremity Wounds. 2021:1-8.
364. Walss M, Anzengruber F, Arafa A, Djamei V, Navarini AA. Implementing Medical Chatbots: An Application on Hidradenitis Suppurativa. Dermatology. 2021;237(5):712-8.
365. Wan B, Ganier C, Du-Harpur X, Harun N, Watt FM, Patalay R, et al. Applications and future directions for optical coherence tomography in dermatology. British Journal of Dermatology. 2021;184(6):1014-22.
366. Wang CY, Tsai T, Chen HM, Chen CT, Chiang CP. PLS-ANN based classification model for oral submucous fibrosis and oral carcinogenesis. Lasers in Surgery and Medicine. 2003;32(4):318-26.
367. Wang HH, Wang YH, Liang CW, Li YC. Assessment of Deep Learning Using Nonimaging Information and Sequential Medical Records to Develop a Prediction Model for Nonmelanoma Skin Cancer. Jama Dermatology. 2019;155(11):1277-83.
368. Wang W, Li MJ, Fan PM, Wang H, Cai J, Wang K, et al. Prototype early diagnostic model for invasive pulmonary aspergillosis based on deep learning and big data training. Mycoses. 2023;66(2):118-27.
369. Wang Y, Zuo K, He ZY, Chen X, Zhang Y, Xie PZ, et al. Real-time burn depth assessment using artificial networks: a large-scale, multicentre study. Burns. 2020;46(8):1829-38.
370. Weber P, Sinz C, Rinner C, Kittler H, Tschandl P. Perilesional sun damage as a diagnostic clue for pigmented actinic keratosis and Bowen's disease. Journal of the European Academy of Dermatology and Venereology. 2021;35(10):2022-6.
371. Wei CM, Adusumilli N, Friedman A, Patel V. Perceptions of Artificial Intelligence Integration into Dermatology Clinical Practice: A Cross-Sectional Survey Study. Journal of Drugs in Dermatology. 2022;21(2):27-32.
372. Wells A, Patel S, Lee JB, Motaparthi K. Artificial intelligence in dermatopathology: Diagnosis, education, and research. Journal of Cutaneous Pathology. 2021;48(8):1061-8.
373. Willem T, Krammer S, Bohm AS, French LE, Hartmann D, Lasser T, et al. Risks and benefits of dermatological machine learning health care applications-an overview and ethical analysis. Journal of the European Academy of Dermatology and Venereology. 2022;36(9):1660-8.
374. Williams HC, McPhee MJ, Layfield CP, Net UKDCT. Celebrating 20 years of the UK Dermatology Clinical Trials Network. Part 1: Developing and delivering high-quality independent clinical trials. Clinical and Experimental Dermatology. 2022;47(6):1048-59.
375. Willingham ML, Spencer S, Lum CA, Sanchez JMN, Burnett T, Shepherd J, et al. The potential of using artificial intelligence to improve skin cancer diagnoses in Hawai'i's multiethnic population. Melanoma Research. 2021;31(6):504-14.
376. Winkler JK, Fink C, Toberer F, Enk A, Deinlein T, Hofmann-Wellenhof R, et al. Association Between Surgical Skin Markings in Dermoscopic Images and Diagnostic Performance of a Deep Learning Convolutional Neural Network for Melanoma Recognition. Jama Dermatology. 2019;155(10):1135-41.
377. Winkler JK, Sies K, Fink C, Toberer F, Enk A, Abassi MS, et al. Collective human intelligence outperforms artificial intelligence in a skin lesion classification task. Journal Der Deutschen Dermatologischen Gesellschaft. 2021;19(8):1178-84.
378. Wongyibulsin S, Ho BKT, Kwatra SG. Embracing machine learning and digital health technology for precision dermatology. Journal of Dermatological Treatment. 2020;31(5):494-5.
379. Wozniacka A, Patrzyk S, Mikolajczyk M. Artificial intelligence in medicine and dermatology. Postepy Dermatologii I Alergologii. 2021;38(6):948-52.
380. Xiao P, Zhang X, Pan W, Ou X, Bontozoglou C, Chirikhina E, et al. The Development of a Skin Image Analysis Tool by Using Machine Learning Algorithms. Cosmetics. 2020;7(3):1-12.
381. Xie PG, Li YY, Deng B, Du CZ, Rui SL, Deng W, et al. An explainable machine learning model for predicting in-hospital amputation rate of patients with diabetic foot ulcer. International Wound Journal. 2022;19(4):910-8.
382. Xiong ML, Pfau J, Young AT, Wei ML. Artificial Intelligence in Teledermatology. Current Dermatology Reports. 2019;8(3):85-90.
383. Xu J, Chen DX, Deng XF, Pan XY, Chen Y, Zhuang XM, et al. Development and validation of a machine learning algorithm-based risk prediction model of pressure injury in the intensive care unit. International Wound Journal. 2022;19(7):1637-49.
384. Yamamichi F, Shigemura K, Yamashita M, Tanaka K, Arakawa S, Fujisawa M. Postoperative wound dealing and superficial surgical site infection in open radical prostatectomy. International Wound Journal. 2016;13(5):692-6.
385. Yang Y, Ge YP, Guo LF, Wu QJ, Peng L, Zhang EJ, et al. Development and validation of two artificial intelligence models for diagnosing benign, pigmented facial skin lesions. Skin Research and Technology. 2021;27(1):74-9.
386. Yang Y, Guo LF, Wu QJ, Zhang ML, Zeng R, Ding H, et al. Construction and Evaluation of a Deep Learning Model for Assessing Acne Vulgaris Using Clinical Images. Dermatology and Therapy. 2021;11(4):1239-48.
387. Yang ZH, Shang JW, Liu CL, Zhang J, Liang YM. Identification of oral precancerous and cancerous tissue by swept source optical coherence tomography. Lasers in Surgery and Medicine. 2022;54(2):320-8.
388. Yap J, Yolland W, Tschandl P. Multimodal skin lesion classification using deep learning. Experimental Dermatology. 2018;27(11):1261-7.
389. Ye HL, Rahul, Dargar S, Kruger U, De S. Ultrasound elastography reliably identifies altered mechanical properties of burned soft tissues. Burns. 2018;44(6):1521-30.
390. Ye JY, Yu C, Husman T, Chen B, Trikala A. Novel strategy for applying hierarchical density-based spatial clustering of applications with noise towards spectroscopic analysis and detection of melanocytic lesions. Melanoma Research. 2021;31(6):526-32.
391. Yeong EK, Hsiao TC, Chiang HK, Lin CW. Prediction of burn healing time using artificial neural networks and reflectance spectrometer. Burns. 2005;31(4):415-20.
392. Yilmaz A, Goktay F, Varol R, Gencoglan G, Uvet H. Deep convolutional neural networks for onychomycosis detection using microscopic images with KOH examination. Mycoses. 2022;65(12):1119-26.
393. Yoo HY, Lee KC, Woo JE, Park SH, Lee S, Joo J, et al. A Genome-Wide Association Study and Machine-Learning Algorithm Analysis on the Prediction of Facial Phenotypes by Genotypes in Korean Women. Clinical Cosmetic and Investigational Dermatology. 2022;15:433-45.
394. Yoo KH, Jeong GJ, Park JH, Park SH, Li KS. Estimation error of the body surface area in psoriasis: a comparative study of physician and computer-assisted image analysis (ImageJ). Clinical and Experimental Dermatology. 2022;47(7):1298-306.
395. Young AT, Vora NB, Cortez J, Tam A, Yeniay Y, Afifi L, et al. The role of technology in melanoma screening and diagnosis. Pigment Cell & Melanoma Research. 2021;34(2):288-300.
396. Young AT, Xiong ML, Pfau J, Keiser MJ, Wei ML. Artificial Intelligence in Dermatology: A Primer. Journal of Investigative Dermatology. 2020;140(8):1504-12.
397. Zaar O, Larson A, Polesie S, Saleh K, Tarstedt M, Olives A, et al. Evaluation of the Diagnostic Accuracy of an Online Artificial Intelligence Application for Skin Disease Diagnosis. Acta Dermato-Venereologica. 2020;100:adv00260.
398. Zakhem GA, Fakhoury JW, Motosko CC, Ho RS. Characterizing the role of dermatologists in developing artificial intelligence for assessment of skin cancer. Journal of the American Academy of Dermatology. 2021;85(6):1544-56.
399. Zhang AJ, Lindberg N, Chamlin SL, Haggstrom AN, Mancini AJ, Siegel DH, et al. Development of an artificial intelligence algorithm for the diagnosis of infantile hemangiomas. Pediatric Dermatology. 2022;39(6):934-6.
400. Zhang S, Wang YZ, Zheng QY, Li JR, Huang JZ, Long X. Artificial intelligence in melanoma: A systematic review. Journal of Cosmetic Dermatology. 2022;21(11):5993-6004.
401. Zhang T, Nie YL. Prediction of the Risk of Alopecia Areata Progressing to Alopecia Totalis and Alopecia Universalis: Biomarker Development with Bioinformatics Analysis and Machine Learning. Dermatology. 2022;238(2):386-96.
402. Zhang Y, Jiang R, Kezele I, Flament F, Elmozino E, Zhang J, et al. A new procedure, free from human assessment, that automatically grades some facial skin signs in men from selfie pictures. Application to changes induced by a severe aerial chronic urban pollution. International Journal of Cosmetic Science. 2020;42(2):185-97.
403. Zhao JW, Jain M, Harris UG, Kose K, Curiel-Lewandrowski C, Kang DK. Deep Learning-Based Denoising in High-Speed Portable Reflectance Confocal Microscopy. Lasers in Surgery and Medicine. 2021;53(6):880-91.
404. Zhao S, Xie B, Li Y, Zhao X, Kuang Y, Su J, et al. Smart identification of psoriasis by images using convolutional neural networks: a case study in China. Journal of the European Academy of Dermatology and Venereology. 2020;34(3):518-24.
405. Zhu XZ, Zheng BW, Cai WY, Zhang J, Lu S, Li XQ, et al. Deep learning-based diagnosis models for onychomycosis in dermoscopy. Mycoses. 2022;65(4):466-72.
406. Zoppo G, Marrone F, Pittarello M, Farina M, Uberti A, Demarchi D, et al. AI technology for remote clinics assessment and monitoring. Journal of Wound Care. 2020;29(12):692-706.
